# Energy and environmental assessment of a traction lithium-ion battery pack for plug-in hybrid electric vehicles

**DOI:** 10.1016/j.jclepro.2019.01.056

**Published:** 2019-04-01

**Authors:** Maria Anna Cusenza, Silvia Bobba, Fulvio Ardente, Maurizio Cellura, Franco Di Persio

**Affiliations:** a University of Palermo, Department of Energy, Information Engineering and Mathematical Models (DEIM), Viale delle Scienze Building 9, Palermo, Italy; b European Commission, Joint Research Centre, Directorate for Sustainable Resources, via Enrico Fermi, 2749, Ispra, VA, Italy; c DIATI – Department of Environment, Land and Infrastructure Engineering, Politecnico di Torino, Corso Duca degli Abruzzi 24, Turin, Italy; d European Commission, Joint Research Centre, Directorate for Energy, Transport and Climate, Energy Storage Unit, Westerduinweg 3, NL-1755 LE, Petten, the Netherlands

**Keywords:** Lithium-ion traction battery, LMO–NMC cell technology, Battery cell material content, Life cycle assessment, Battery recycling process

## Abstract

Traction batteries are a key factor in the environmental sustainability of electric mobility and, therefore, it is necessary to evaluate their environmental performance to allow a comprehensive sustainability assessment of electric mobility. This article presents an environmental assessment of a lithium-ion traction battery for plug-in hybrid electric vehicles, characterized by a composite cathode material of lithium manganese oxide (LiMn_2_O_4_) and lithium nickel manganese cobalt oxide Li(Ni_x_Co_y_Mn_1-x-y_)O_2_. Composite cathode material is an emerging technology that promises to combine the merits of several active materials into a hybrid electrode to optimize performance and reduce costs. In this study, the environmental assessment of one battery pack (with a nominal capacity of 11.4 kWh able to be used for about 140,000 km of driving) is carried out by using the Life Cycle Assessment methodology consistent with ISO 14040. The system boundaries are the battery production, the operation phase and recycling at the end of life, including the recovery of various material fractions. The composite cathode technology examined besides a good compromise between the higher and the lower performance of NMC and LMO cathodes, can present good environmental performances.

The results of the analysis show that the manufacturing phase is relevant to all assessed impact categories (contribution higher than 60%). With regard to electricity losses due to battery efficiency and battery transport, the contribution to the use phase impact of battery efficiency is larger than that of battery transport. Recycling the battery pack contributes less than 11% to all of the assessed impact categories, with the exception of freshwater ecotoxicity (60% of the life cycle impact). The environmental credits related to the recovery of valuable materials (e.g. cobalt and nickel sulphates) and other metal fractions (e.g. aluminium and steel) are particularly relevant to impact categories such as marine eutrophication, human toxicity and abiotic resource depletion.

The main innovations of this article are that (1) it presents the first bill of materials of a lithium-ion battery cell for plug-in hybrid electric vehicles with a composite cathode active material; (2) it describes one of the first applications of the life cycle assessment to a lithium-ion battery pack for plug-in hybrid electric vehicles with a composite cathode active material with the aim of identifying the “hot spots” of this technology and providing useful information to battery manufacturers on potentially improving its environmental sustainability; (3) it evaluates the impacts associated with the use phase based on primary data about the battery pack's lifetime, in terms of kilometres driven; and (4) it models the end-of-life phase of the battery components through processes specifically created for or adapted to the case study.

## Highlights

• A bill of materials of an LMO-NMC Li-ion battery cell is presented.
• A from-cradle-to-grave life cycle assessment of the battery pack is carried out.
• The operation phase impacts are estimated based on real battery cells lifetime.
• The recycling process of the LMO-NMC cells is tailored to the specific case – study.
• A scenario analysis highlights the influence of the assumptions on the results.

## Abbreviations

ADP: abiotic depletion potential
BEV: battery electric vehicle
BMS: battery management system
BoM: bill of materials
CED: cumulative energy demand
CMC: carboxymethyl cellulose
E_FW_: freshwater ecotoxicity
EoL: end of life
EU: European Union
EU_F_: freshwater eutrophication
EU_M_: marine eutrophication
EU_T_: terrestrial eutrophication
EV: electric vehicle
FU: functional unit
GWP: global warming potential
HT-ce: human toxicity – cancer effect
HT-nce: human toxicity – no cancer effect
ICE: internal combustion engine
IEA: International Energy Agency
IR-hh: ionizing radiation – human health
JRC: Joint Research Centre
LCA: life cycle assessment
LCI: life cycle inventory
LCIA: life cycle impact assessment
LFP: LiFePO_4_
Li-ion: lithium-ion
LMO: LiMn_2_O_4_
NMC: Li(NixCoyMn1-x-y)O_2_
NMP: N-methyl-2-pyrrolidone
ODP: ozone depletion potential
PAA: polyacrylic acid
PE: polyethylene
PEF: product environmental footprint
PHEV: plug-in hybrid electric vehicle
PM: particulate matter
POFP: photochemical ozone formation potential
PP: polypropylene
PVDF: polyvinylidene fluoride
PWB: printed wiring board
RES: renewable energy source

## 1 Introduction

Electricity is currently one of the most relevant energy carriers used in decarbonisation of the energy sector in terms of either building applications (Cellura et al., 2017, 2015; Finocchiaro et al., 2016; Ortiz et al., 2014) or transportation (Spencer et al., 2017). In particular, as an energy carrier for vehicle propulsion, electricity offers the possibility of replacing fossil fuels used in internal combustion engine (ICE) vehicles with renewable energy sources (RESs), allowing considerable reductions in CO_2_ emissions from the automotive sector (Zackrisson et al., 2010).

According to the International Energy Agency (IEA), the number of electric vehicles (EVs) will increase from 2 million units in 2016 to 56 million by 2030 (International Energy Agency, 2017).^1^ In this context, understanding the system-wide trade-offs of replacing ICE vehicles with EVs is paramount and requires a life cycle perspective (Ellingsen et al., 2017). The life cycle assessment (LCA) is a standardized methodology (ISO 14040) widely adopted by the scientific community to assess the environmental impacts of products and services from such a perspective (ISO, 2006a, 2006b).

The preferred technology for traction batteries is lithium-ion (Li-ion) chemistry (Ellingsen et al., 2014; European Energy Agency, 2016; Nam et al., 2009; Schexnayder et al., 2001; Van den Bossche et al., 2006; Vazquez et al., 2010). Different types of Li-ion batteries, using various compositions of both the cathode and the anode, are currently available. However, whereas the anode is usually made of graphite (natural or synthetic) (Steen et al., 2017), there are greater differences in the active materials used in the cathode, which is usually made of LiMn_2_O_4_ (LMO) (spinel), LiFePO_4_ (LFP) or Li(Ni_x_Co_y_Mn_1-x-y_)O_2_ (NCM) (Steen et al., 2017). The last one may also be combined with LMO in a composite cathode (LMO–NMC). The concept of composite electrodes promises to combine the merits of several active materials into a hybrid electrode for optimized performances (Dubarry et al., 2011; Fergus, 2010; Zubi et al., 2018). In fact, LMO (spinel), with a three-dimensional structure and Li-ion diffusion, offers high rate capability and good structural stability (Manthiram, 2017), as well as relatively low production costs (Dubarry et al., 2011). However, it has a relatively small capacity (around 100–150 Wh/kg) and a cycle life of about 300–700 cycles (Battery University., 2018), and it will degrade if manganese (Mn^2+^) dissolves in the electrolyte and is subsequently deposited on the anode in the charge regime. NMC has a greater capacity (150–220 Wh/kg) and a cycle life of about 1000–2000 cycles (Battery University., 2018; Cobalt Institute, 2018) but it can suffer from structural and/or chemical instabilities during cycling (Manthiram, 2017). The LMO–NMC composite cathode is a compromise to provide an electrode that exhibits good performance in terms of capacity and structure stability: the LMO part of the battery provides a high boost of current on acceleration while the NMC part gives a long driving range (Battery University., 2018). Moreover, this chemistry can guarantee a lower price and less vulnerability to supply disruption because of its lower levels of cobalt, which is the most costly item (Chagnes and Pospiech, 2013). Various EVs, such as the Nissan Leaf, Chevy Volt and BMW i3, have adopted the LMO–NMC chemistry (Battery University., 2018).

This article reports on an LCA carried out to examine the life cycle environmental impacts of an Li-ion plug-in hybrid EV (PHEV)^2^ battery pack made of an LMO–NMC composite cathode and to identify the contribution of each life cycle phase. In addition, since one of the added values of this composite cathode is the smaller amount of cobalt, which is an expensive part of the battery and also recognized as “critical” for Europe (Cobalt Institute, 2018) (Blengini et al., 2017a, Blengini et al., 2017b; European Commission, 2017), the authors estimate the cobalt content of the battery cell examined.

The bill of materials (BoM) of the LMO–NMC cell was compiled based on primary data from laboratory analysis, which is an important innovation of this article. In fact, as highlighted by Peters et al. (2017) and Ellingsen et al. (2017), few studies have so far provided an original and detailed life cycle inventory (LCI). Specifically, among the 79 LCAs on Li-ion batteries examined by Peters et al. (2017), in only nine publications did the authors provide their own inventory data (Dunn et al., 2012a; Ellingsen et al., 2014; Gaines and Cuenca, 2000; Hischier et al., 2007; Majeau-Bettez et al., 2011; Notter et al., 2010; Rydh and Sandén, 2005; U.S. EPA, 2013; Zackrisson et al., 2010). The literature analysis showed that few data on traction Li-ion batteries are available and those that were available were systematically used in various LCAs. Therefore, to increase the assessment's reliability, it was important to use primary industry data as far as possible.

The BoM of the LMO–NMC cell has been created for the first time for this study and is an important contribution of this study to the state of the art. In fact, although existing LCA studies cover Li-ion traction batteries that have different active cathode materials, such as LMO (Notter et al., 2010; Richa et al., 2015), LFP (Majeau-Bettez et al., 2011; Zackrisson et al., 2010) and NMC (Ellingsen et al., 2014; Majeau-Bettez et al., 2011), to the authors’ knowledge the only environmental assessment of an LMO–NMC traction battery for battery electric vehicles (BEVs) was carried out by Kim et al. (2016). However, Kim et al. (2016) did not provide a contribution analysis of cell materials and analysed only one impact category (i.e. global warming potential (GWP)).

Secondary data from literature studies were used to complement the inventory of other battery pack components (i.e. the battery management system (BMS), cooling system and packaging), as the authors had access to the cells only and not to the whole battery pack. However, this did not lessen the relevance of the results presented here because, as highlighted in several LCAs (Ellingsen et al., 2014; Kim et al., 2016; Majeau-Bettez et al., 2011; Notter et al., 2010), cells are the components responsible for the greatest impacts in battery pack production.

The impacts associated with the use phase are based on primary data about the battery pack lifetime (in terms of effective kilometres driven, i.e. 136,877 km) of the battery cells examined, and these have been estimated from the literature and from technical specifications set out in the Mitsubishi PHEV Outlander catalogue.

At the end of a battery's life, it is assumed that the components are dismantled and treated for recycling. In particular, detailed data on the end-of-life (EoL) processes tailored to the specific case study are presented and the potential environmental impacts of and benefits from the production of secondary raw materials are identified. With particular reference to the battery cells, the recycling was modelled in accordance with recent studies, such as on the product environmental footprint category rules (PEFCRs) on rechargeable batteries (Recharge, 2018); research on the recyclability of different materials (Chancerel and Marwede, 2016); and values from specialized industries sectors (UMICORE, 2018). More specifically, a pyrometallurgical recycling treatment followed by a hydrometallurgical one is considered, and the potential environmental credits resulting from the recycling of recoverable products, depending on the composition of the battery cell examined, are assessed. This is an important contribution of the study as, to the authors' knowledge, most previous LCAs of Li-ion batteries have not provided a detailed analysis of recycling in terms of environmental impacts and credits.

Finally, the article discusses how the study assumptions affected the results obtained.

The main innovations of the article are: (1) it presents a first BoM of an LMO–NMC Li-ion battery cell for PHEVs compiled using both primary and secondary data (these data can be used to create an International Reference Life Cycle Data System-compliant database to be published in the JRC Life Cycle Data Network); (2) it provides a set of life cycle energy and environmental indicators and identifies the “hot spots” of LMO–NMC battery technology that could provide useful information for battery manufacturers looking to improve sustainability; (3) it evaluates the impacts associated with the use phase based on primary data about the battery pack's lifetime, in terms of kilometres driven; and (4) it estimates the potential impacts of and benefits from battery recycling at EoL through processes specifically created for or adapted to the specific case study.

## 2 LCA of Li-ion traction batteries: state of the art

Several LCA studies on traction Li-ion batteries suitable for applications in PHEV and BEV are available in the literature. In this section, the authors analyse some of these to highlight the LCI data sources for the LCA, the Li-ion battery technologies examined and the battery EoL modelling.

Although a comparison of studies is complex because of differing assumptions, in terms of both method (e.g. system boundaries) and battery characteristics (e.g. cathode and anode composition), in this article the comparison of results focuses on global warming potential, since this is the only impact category reported in all of the reviewed studies and, additionally, it is estimated using the same impact assessment method (IPCC, 2007). Moreover, the comparison between the results obtained and the results available in the literature is based only on the production phase, since this is generally the phase more precisely assessed and less affected by the variability of assumptions regarding the operation and EoL stages (Longo et al., 2014).

The main battery characteristics, system boundaries and the impact per kWh reported in the studies examined are listed in Table 1.

**Table 1:** LCA studies on traction Li-ion batteries.

References	Battery characteristics	System boundaries	Battery data sources	GWP associated with the production phase per kWh of battery energy capacity
Richa et al., 2017	BEV Li-ion traction battery; cathode: LMO; energy capacity: 24 kWh; weight: 223 kg; battery efficiency: 95%	Battery production, use in the EV, re-manufacturing, second use in stationary ESS, recycling (hydrometallurgical + pyrometallurgical processes)	Literature data (Argonne National Laboratory, 2011)	58.3 kgCO_2eq_
Kim et al. (2016)	Li-ion traction battery for BEV; cathode: LMO–NMC; energy capacity: 24 kWh; weight: 300 kg	Battery production	Primary data from battery industry	140 kgCO_2eq_
Faria et al. (2014)	Li-ion traction battery for BEV; cathode: LMO; energy capacity: 24 kWh; weight: 300 kg	Battery production, use in the EV, re-manufacturing, second use in stationary ESS, recycling (hydrometallurgical process)	Literature data (Notter et al., 2010)	70.9 kgCO_2eq_
Ellingsen et al. (2014)	BEV Li-ion traction battery; cathode: NMC; energy capacity: 26.6 kWh; weight: 253 kg; battery efficiency: 95–96%	Battery production	Own primary data from battery manufacturer + literature data (Majeau-Bettez et al., 2011)	172 kgCO_2eq_ (cell assembly 586 MJ/kWh)240 kgCO_2eq_ (cell assembly 960 MJ/kWh)487 kgCO_2eq_ (cell assembly 2318 MJ/kWh)
Majeau-Bettez et al. (2011)	BEV and PHEV Li-ion traction battery, cathode: LFP, NMC	Battery production, use in the EV	Own primary data + literature data (Gaines and Cuenca, 2000); (Schexnayder et al., 2001); (Rydh and Sandén, 2005))	NMC/LFP: 200/250 kgCO_2eq_
US EPA, 2013	1.40 kWh BEV Li-ion traction battery, cathode: LMO, LFP, NMC;	Battery production, use in the EV, recycling (hydrometallurgical, pyrometallurgical, direct recycling processes)	Own primary data + literature data (Notter et al., 2010); (Majeau-Bettez et al., 2011)	112 kgCO_2eq_
	2.11.6 kWh PHEV Li-ion traction battery, cathode: LMO, LFP, NMC			
Zackrisson et al. (2010)	1. PHEV Li-ion traction battery, cathode: LFP (NMP as a solvent)	Battery production; use phase; battery transport to recycling	Literature data (Gaines and Cuenca, 2000), laboratory tests (Swerea IVF), Saft's report (2008)	266 kgCO_2eq_ (NMP as a solvent); 166 kgCO_2eq_ (water as a solvent)
	2. PHEV Li-ion traction battery, cathode: LFP (water as a solvent)			
Notter et al. (2010)	BEV Li-ion traction battery, cathode: LMO; battery capacity: 34.2 kWh	Production, maintenance, EoL and operation of the Li-ion battery and maintenance and EoL of the road, glider, train and car	Own primary data (battery produced by Kokam Company^a^)	52.6 kgCO_2eq_

a http://kokam.com/.

The literature examined highlights the difficulty of carrying out an LCA of Li-ion battery production when relying on only primary inventory data for foreground processes, i.e. those processes that the decision maker or the product's owner can influence directly (Frischknecht et al., 1998). Therefore, a deeper analysis of the battery components is needed, paying particular attention to the battery cells. As the review shows, these are the components mainly responsible for the battery's environmental impacts. Among the eight studies examined, four considered LMO technology, three considered NMC and LFP technologies, and only one study referred to LMO–NMC technology. However, this last did not provide a contribution analysis of the cell materials.

Regarding the operation phase, all of the LCA studies on traction Li-ion batteries examined factored in that the battery pack would need to be replaced after 150,000–160,000 km based on automotive industry warranties (Ahmadi et al., 2017; Faria et al., 2014; Hawkins et al., 2013; Richa et al., 2015) or on the authors’ assumption (Girardi et al., 2015; Hooftman et al., 2018; Szczechowicz et al., 2012; Wu et al., 2018; Zackrisson et al., 2010). There is no evidence in the LCA studies of experimental data about battery lifetime.

Regarding the modelling of the EoL phase, it was observed that pyrometallurgical or hydrometallurgical processes were assumed in the studies reviewed. However, among the LCAs examined (Casals et al., 2017; Faria et al., 2014; Notter et al., 2010; U.S. EPA, 2013), only Richa et al. (2017b) provided a detailed description of the recycling process, modelled on the basis of a pyrometallurgical process for 50% of the EoL Li-ion cells and a hydrometallurgical one for the remaining 50%, and of the related impacts and benefits. Furthermore, several LCAs did not include the EoL phase in the analysis because of the greater uncertainty (mainly due to lack of data) (Ahmadi et al., 2014; Ellingsen et al., 2014; Kim et al., 2016; Majeau-Bettez et al., 2011; Zackrisson et al., 2010). The current article considers available life cycle datasets (as in the Ecoinvent database) that have been updated in accordance with the abovementioned studies (Chancerel and Marwede, 2016; Recharge, 2018; UMICORE, 2018), tailored to the materials actually used in the battery chemistry assessed in the article and designed to take into account the recycling of additional components (e.g. BMS, cooling system, battery packaging).

The following sections therefore provide a detailed analysis of an LMO–NMC battery inventory based on primary data. Moreover, an analysis of a potential EoL scenario for batteries is presented and discussed.

## 3 Life cycle assessment

### 3.1 Goal and scope definition

The goals of the study are:

• to provide LCI data on an LMO–NMC traction battery cell;
• to estimate the potential life cycle environmental impacts of an LMO–NMC PHEV battery pack and to assess the contribution of each life cycle phase;
• to estimate the cobalt content of the LMO–NMC battery cell technology examined;
• to assess the potential environmental impacts of and benefits from the production of secondary raw materials at the battery's EoL;
• to assess how the study assumptions affected the results obtained.

The authors apply an attributional LCA approach in accordance with the international standards of series ISO 14040 (ISO, 2006a, 2006b).

**Fig. 1:**
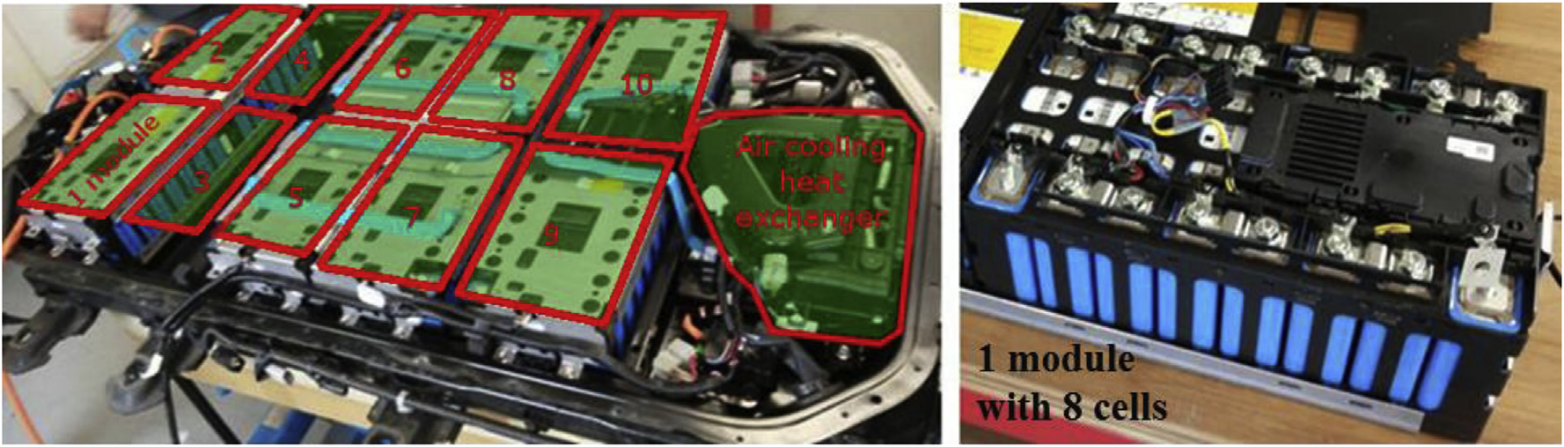
Left, battery pack; right, battery cells grouped into one module.

According to Ellingsen et al. (2014), battery components are classified as battery cells, BMS, cooling system and battery packaging. The battery cells are grouped into modules. Fig. 1 shows the battery pack examined with an indication of the placement of the 10 modules, with a closer view of one module with its eight cells. The approximate location of the air cooling heat exchanger is also shown, while the BMS is embedded and distributed in the pack.

The cell is the electrochemical unit of the battery. It contains the electrodes (cathode and anode), the separator and the electrolyte packet enclosed in the cell case (detailed in Section 2 of the supplementary material). The BMS manages the battery cells to ensure that they operate within safe parameters, and it includes electronics boards, fasteners and high- and low-voltage systems. The cooling system ensures that the battery cells work in a safe-operating temperature range. Finally, the battery packaging serves as a structural support.

The case study analyses a Mitsubishi Outlander LEV40 LMO–NMC PHEV battery pack, purchased by the JRC from a car dealer, who replaced the battery pack from a customer's EV after about 140,000 km (specifically 136,877 km) (Bobba et al., 2018a) driven in electric mode as much as possible. The main characteristics of the battery under investigation are detailed in Table 2.

**Table 2:** Technical characteristics of the battery.

Characteristics	Battery pack
Nominal voltage (V)	300
Nominal capacity (Wh)	11,400
Number of cells	80 (grouped in 10 modules)
Type of cell	Prismatic
Weight of the cells (W_c_) (kg)	105.6
Weight of the battery pack (W_b_) (kg)	175

The functional unit (FU) selected as the reference for the LCA analysis is one LMO–NMC battery pack with a nominal capacity of 11.4 kWh, which guaranteed 136,877 km of driving for a passenger car weighing 1860 kg before the battery capacity reduced about 81.31% (Bobba et al., 2018a).

The following phases were included in the analysis:

• the production phase (including raw material supply, material production, cell and battery pack assembly, transport and infrastructure);
• the use phase in the PHEV (including electricity consumed as a result of the battery's internal efficiency and by carrying the weight of the battery);
• the EoL phase (including the recycling of each component).

The impact assessment was based on the methods recommended by the European Product Environmental Footprint (PEF) (European Commission, 2013), which provide a large set of environmental indicators consistent with the sustainability objective of avoiding burden-shifting among impact categories (Hauschild et al., 2017). Because energy consumption is highly relevant to the evaluation of the studied system, the PEFCRs were complemented by the cumulative energy demand (CED) method for energy impact estimation (Frischknecht et al., 2007). Moreover, in accordance with Bobba et al. (2016) and Latunussa et al. (2016), the land use and water resource depletion impact categories were excluded (as a result of the low availability and high uncertainty of LCI data). The abiotic depletion potential was calculated only for mineral resources (to avoid overlapping with the CED impact category).

**Fig. 2:**
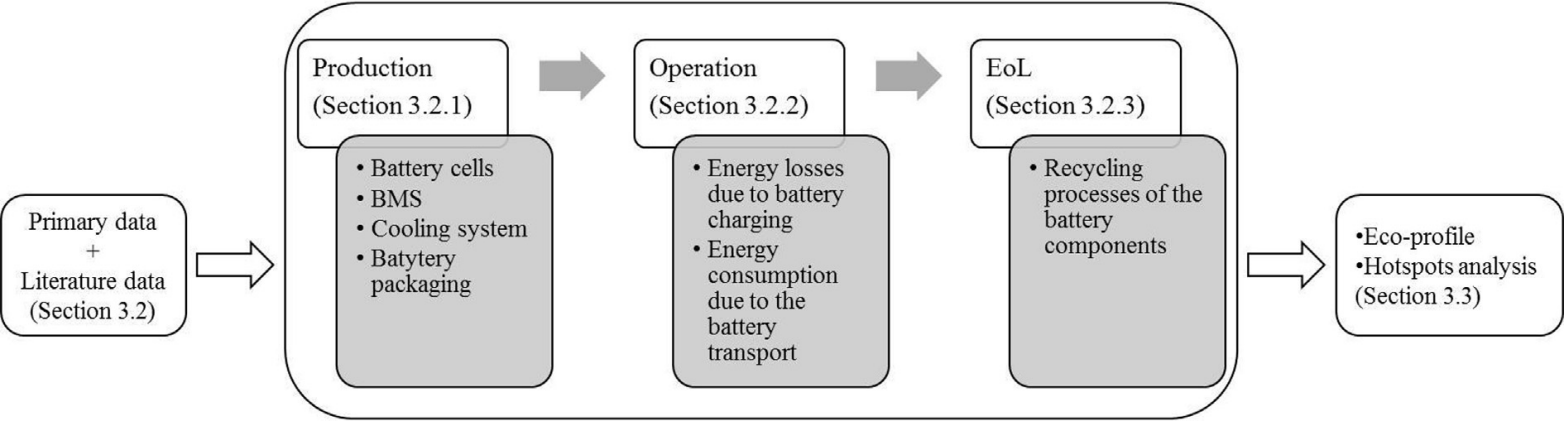
LCA modelling scheme.

Fig. 2 shows the LCA modelling scheme.

### 3.2 Life cycle inventory

In general, both primary and secondary data were used for the inventory of the battery pack. The LCI of the cells was compiled based on the dismantled battery pack studied (see Section 3.2.1), whereas Majeau-Bettez et al. (2011) and Ellingsen et al. (2014) were the main sources of data for the upstream processes for the production of each cell component, the energy required for their assembly and the inventory of BMS, cooling system and battery packaging.

Virgin materials were assumed for raw material inputs (e.g. aluminium, copper, plastics). Recycled materials at EoL were assumed to displace virgin materials^3^. The eco-profiles of materials and energy sources used to produce the battery components were based on the Ecoinvent 3 database (Wernet et al., 2016). It was assumed that the production phase of the battery components occurred in Japan, and thus the Japanese electricity mix was used. The amount of electricity needed to assemble the cells and the battery pack was inferred from Ellingsen et al. (2014) 4. The operation phase and the EoL phase were assumed to take place in Europe, and thus the average European electricity mix was used to model these life cycle stages. Transport and infrastructure requirements during the manufacturing stage of the components were based on Ellingsen et al. (2014).

In the following section, the authors describe the procedure and the assumptions used to compile the BoM of the LMO–NMC battery cell, by combining primary data (Table 3) with literature data.

**Table 3:** Material breakdown of a fresh LMO–NMC/graphite cell as determined by dismantling and further analysis.

LMO-NMC cell (total weight before opening: 1396.2 g)	% in weight (%)	Fraction/g	Accuracy (g)
Steel: external case, connectors	21.47	299.8	±2
Al: current collectors, electrode foils	3.74	52.2	±2
Cu: current collectors, electrode foils	10.03	140.0	±6
Polymer: wrapping, tapes, separator	5.99	83.6	±2
Anode active material: graphite	10.17	142.0	±12
Binder	2.68	37.4	±6
Cathode active material: LMO-NMC	27.47	383.5	±20
Carbon black in the cathode	3.38	47.2	±32
Electrolyte	13.75	192.0	±20
Uncounted materials lost in cutting/drilling/handling (steel, polymer, Cu, Al, active materials)	1.32	18.5	±5

#### 3.2.1 Cell material breakdown and life cycle inventory

A new LMO–NMC cell was disassembled in a glove box in an inert argon atmosphere, and a material breakdown analysis was performed. The process is described in Section 3 of the supplementary material. The BoM resulting from dismantling and further analysis is detailed in Table 3.

The BoM of the battery cell components and the type of data source (i.e. primary or secondary) used in the cell modelling are shown in Table 4. The detailed inventory for LiNi_0.4_Co_0.2_Mn_0.4_O_2_ is not reproduced here, as it is reported in the supporting information of the original studies carried out by Majeau-Bettez et al. (2011) and Ellingsen et al. (2014).

**Table 4:** BoM of the LMO–NMC cell and main assumptions for cell modelling.

Cell components	Composition	Mass (g)
Anode		282.94^∗∗∗^ (P)
The specific composition of the negative active material and of the binder was unknown, so they were taken from a study (Ellingsen et al., 2014). The required amounts were determined during battery cell dismantling. In anode manufacturing, a solvent was used to give the mixture a slurry texture. After the negative paste was applied to the current collector, the solvent evaporated. The information about solvent is not available, so its composition was modelled in accordance with studies (Ellingsen et al., 2014; Gaines and Cuenca, 2000; Majeau-Bettez et al., 2011). The required amount was taken from Ellingsen et al. (Ellingsen et al., 2014).	Negative current collector: copper (P^∗^)	113.48 (P)
	Negative active material: synthetic graphite (L**) (Ellingsen et al., 2014)	162.24 (P)
	Binder: 0.5 polyacrylic acid (PAA) + 0.5 carboxymethyl cellulose (CMC) (L)	7.22 (P)
	Solvent: N-methyl-2-pyrrolidone (NMP) (L)	159.8 (L)
Cathode		502.82^∗∗∗^ (P)
The specific composition of the positive active material was provided by the battery manufacturer. The active cathode material composition for the analysed battery was modelled as 52% of LiMn_2_O_4_ (LMO) and 48% of Li(Ni_0.4_Co_0.2_Mn_0.4_)O_2_ (NMC). The LMO inventory was taken from the Ecoinvent database, while the NMC inventory was from Majeau-Bettez et al. (2011) and Ellingsen et al. (2014). Based on Ellingsen et al. (2014), the binder was assumed to be PVDF, with the required amounts determined during battery cell dismantling. Similarly to the negative electrode paste, in the positive electrode paste manufacturing NMP was considered to be the solvent and the required amount was taken from Ellingsen et al. (2014).	Positive current collector: aluminium (P)	40.36 (P)
	Positive active material: LMO (P/L)	217.45 (P)
	Positive active material: NMC (P/L)	200.73 (P)
	Binder: polyvinylidene fluoride (PVDF) (L)	19.68 (P)
	Carbon (P)	24.6 (P)
	Solvent: NMP (L)	189.6 (L)
Electrolyte		170.58 (P)
The specific composition of the electrolyte was not detected during cell dismantling. Therefore, it was modelled in accordance with the literature (Ellingsen et al., 2014; Gaines and Cuenca, 2000; Kim et al., 2016; Notter et al., 2010). The amount of electrolyte per battery cell was determined in the laboratory.	Lithium hexafluorophosphate (LiPF_6_) (L)	150.11 (L)
	Ethylene carbonate (C_3_H_4_O_3_) (L)	20.47 (L)
Separator		67.4 (P)
The specific material composition of the separator was not determined, so it was modelled in accordance with Nelson et al. (2011). The weight was determined in the laboratory.	Polypropylene, granulate (PP) (L)	53.92 (L)
	Polyethylene, granulate (PE) (L)	13.48 (L)
Cell case		372.47 (P)
The cell case was made of steel. It contained the anode and cathode soaked with electrolyte and folded together with the separator in two jelly rolls that were properly connected to the two external negative and positive tabs. The composition of the case was obtained by combining the data determined in the laboratory with the LCI by Ellingsen et al. (2014).	Aluminium (P/L)	11.77 (P)
	Copper (P/L)	26.38 (P)
	Packaging film (P/L)	7.23 (P)
	Polyethylene terephthalate, granulate (P/L)	5.36 (P)
	Polypropylene, granulate (PP) (L)	22 (P)
	Steel (P/L)	299.72 (P)
Total		1396.20^∗∗∗^

*Primary data, **Literature data, ***The amounts of NMP used in cathode and anode manufacturing are not included in the total.

#### 3.2.2 The battery operation phase

According to several literature studies (Longo et al., 2014; Matheys and Autenboer, 2005; Zackrisson et al., 2010), the battery operation phase accounts for electricity losses in the battery during use (i.e. to power the car for transport) and the extra electricity needed by the vehicle to carry the battery. The electricity consumed by the battery during operation is calculated using the following assumptions:

• the PHEV runs on electric mode for 75% (El_drm_) and on petrol mode for the remaining 25% (P_drm_) (Zackrisson et al., 2010);
• the car consumes 0.192 kWh electricity per kilometre in electric mode (CEl_drm_) (Mitsubishi Motors, 2018);
• 30% of the vehicle's energy consumption can be related to battery transport (30% weight–energy relationship) (CEl_w_).

The traction Li-ion battery examined is driven for about 140,000 km (D_dr_) during the PHEV's lifetime at 80% maximum DoD and with 95% charging efficiency (η_c_) (Bobba et al., 2018a). The kerb weight of the car is inferred from the technical specification reported in the Mitsubishi catalogue for Outlander PHEV, i.e. 1860 kg (Mitsubishi Motors, 2018).

Electricity losses due to internal battery efficiency (El_be_) are calculated using the following equation (Eq. (1)):

(1) $$El_{be}=D_{dr}\cdot El_{drm}\cdot CEl_{drm}\cdot \left(1-\eta_{c}\right)=136,877\;km\cdot 75\%\cdot 0.192\frac{kWh}{km}\cdot 5\%=986\;kWh$$

The extra electricity needed to carry the battery (El_bw_) is calculated using the following equation (Eq. (2)):

(2) $$El_{bw}=\frac{W_{b}}{W_{c}}\cdot CEl_{w}\cdot \frac{CEl_{drm}}{\eta_{c}}\cdot D_{dr}\cdot El_{drm}=\frac{175\;\text{kg}}{1860}\cdot 30\%\cdot \frac{0.192\;kWh}{95\%}\cdot 136,877\;km\cdot 75\%=586\;kWh$$

#### 3.2.3 Battery end of life

In accordance with the Waste Batteries Directive (Directive, 2006/66/EC) (EU, 2006), when traction batteries in EVs reach their EoL, they have to be properly collected and recycled. In this section, the authors assess the environmental impacts of and the potential environmental credits associated with battery pack recycling. In accordance with the PEFCRs on rechargeable batteries (Recharge, 2018), the battery pack was assumed to be dismantled at EoL to separate the main components and maximise the recovery of the various material fractions. All of the manufacturing input materials (aluminium, copper, steel, etc.) were modelled as 100% of primary, which means that no environmental credits were considered to arise from recycled material content. Potential benefits from material recycling were credited to the EoL stage (in terms of “avoided primary materials”).

Regarding the battery cells, in accordance with Chagnes and Pospiech (2013) and PEFCRs on rechargeable batteries (Recharge, 2018), it was assumed that these were recycled through a pyrometallurgical process, since this is commonly used in Europe for battery recycling (Mathieux et al., 2017; Swain, 2017; Tamiang and Angka, 2014). The concentrated and relatively clean metal alloy and the slag obtained are then treated through a hydrometallurgical process to extract valuable metals from both the metal alloy and the slag (Recharge, 2018; UMICORE, 2018). Pyrometallurgical recovery relies on high-temperature smelting to recover the metals and other materials (U.S. EPA, 2013). Through smelting, the metal oxides are converted to their metallic form, a molten metal alloy, containing, in the case of the battery cell examined, nickel, cobalt, copper and steel (Dunn et al., 2012a; Kushnir, 2015; Lebedeva et al., 2016; Mancini et al., 2013). This process does not allow the recovery of graphite, plastic materials, aluminium, lithium or manganese. The last three elements are entrained in the slag produced during the process (Dunn et al., 2012a). The plastic materials are burned and not recyclable (Dunn et al., 2012a; Elibama, 2014). Moreover, carbon black, binder, CMC, PAA, electrolyte and graphite (which account for 37.8% of the total mass of the cells) are currently not recyclable and therefore lost during recycling (burned, evaporated or dispersed in the slag) (Meshram et al., 2014; Richa et al., 2014). It was assumed that the metal alloy and the slag resulting from the pyrometallurgical process, equal to about 55% of the total weight of the cells, were refined with a hydrometallurgical process to recover metal sulphate, which can be used again to manufacture batteries’ active materials (Recharge, 2018). The recoverable materials, depending on the composition of the battery cell examined, may include cobalt, nickel and manganese sulphates, copper and steel. Then, for the LCA model, the environmental credits for avoiding the production of an equivalent amount of the recovered materials were considered.

The inventories for the pyrometallurgical and hydrometallurgical treatments were based on the Ecoinvent database (Wernet et al., 2016). Regarding the plastic in the cell, the original Ecoinvent pyrometallurgical process was modified according to the Batteries 2020 project (Batteries 2020 Project., 2016) and Fisher and Wallén (2006); therefore, instead of the original average treatment process for plastic mixtures that considers disposal in landfill, incineration and use as an alternative fuel and raw material in clinker production, only incineration is considered for plastics.

The BMS, the cooling system and the battery packaging were further dismantled into, for example, metal fractions, plastic fractions, printed wiring board (PWB) fractions, used cable, plastic materials and electronic scraps through a combination of manual dismantling and mechanical separation and sorting (Wernet et al., 2016). It was hypothesized that the copper fraction was recycled in a non-ferrous metal smelter, that the steel fraction was recycled in an electric arc furnace and that the process included steel making and casting. Finally, for the recycling of the aluminium fraction, an average European melting, alloying and casting technology was assumed. The inventories for the EoL of the battery cells, the BMS, the packaging and the cooling system are shown in Table 5, while Table S1 (supplementary material) lists the recycling rates of the recovered materials (Chancerel and Marwede, 2016).

**Table 5:** Inventory data used for the battery cells, BMS, packaging and cooling system EoL treatment modelling.

Reference product	1 kg of cell	1 kg of molten metal alloy + slag	1 kg of BMS	1 kg of packaging	1 kg of cooling system	Ecoinvent processes used for the EoL treatment
Inputs from nature						

Water (m^3^)	1.00E–03	7.2E–04				

Inputs from technosphere						

Aluminium scrap preparation (kg)			0.04	0.36	0.91	Aluminium scrap, post-consumer, prepared for melting; treatment of aluminium scrap, post-consumer, by collecting, sorting, cleaning, pressing
Blister copper conversion facility (p)	5.00E–10		–	–	–	Blister copper conversion facility
Copper scrap preparation (kg)			0.08	0.01		Copper, treatment of scrap by electrolytic refining
Sodium hydroxide (kg)	0.35		–	–	–	Sodium hydroxide, without water, in 50% solution state
Sulfuric acid (kg)		0.23				Sulfuric acid production
Chemical inorganic		0.025				Chemical inorganic
Lime, hydrated		0.116				Lime, hydrated, packed
Chemical factory, organic		4.0E–10				Chemical factory, organic
Steel scrap preparation (kg)			0.41	0.36	0.02	Iron scrap, sorted, pressed, sorting and pressing of iron scrap
Electricity, medium voltage (kWh)	0.80	0.14	0.18	0.25	0.27	Electricity, medium voltage
Electricity, high voltage (kWh)			0.09	0.01		Electricity, high voltage
Heat, natural gas (MJ)			0.30	2.96	7.51	Heat production, natural gas, at boiler condensing modulating >100 kW
Heat, heavy fuel (MJ)			0.02	0.04	0.47	Heat production, heavy fuel oil, at industrial furnace 1 MW
Heat, hard coal (MJ)			0.50	0.10		Heat production, at hard coal industrial furnace 1–10 MW
Emissions to air (for details about emissions to air please consult Ecoinvent 3 database)						

Emissions to water (for details about emissions to air please consult Ecoinvent 3 database)						

Output to technosphere (waste for further treatment)						
Electronic scrap (kg)	–		0.14	–	–	Treatment of electronics scrap from control unit
Non-Fe-Co metals^∗∗^ (kg)		0.18^∗^	–	**–**	**–**	Non-Fe-Co metals, treatment of used Li-ion battery, hydrometallurgical processing
PWB (kg)	–		0.14	–	–	Used printed wiring boards, treatment of scrap printed wiring boards, shredding and separation
Used cable (kg)	–		0.14	–	–	Used cable
Waste graphical paper (kg)		0.065				Waste graphical paper
Waste gypsum		0.339				Waste gypsum
Plastic material in the cells	0.07^∗^	–^∗∗∗^	–	–	–	Waste plastic to municipal incinerator
Plastic materials (kg)	–		0.04	0.24	–	Waste plastic, mixture

Avoided product						

Aluminium (kg)			0.04	0.35	0.89	Aluminium, primary, ingot
Cobalt sulphate (kg)		0.04				Cobalt sulphate (Majeau-Bettez et al., 2011)
Copper (kg)		0.10	0.08	0.01	–	Copper production, primary
Nickel sulphate (kg)		0.07				Nickel sulphate (Majeau-Bettez et al., 2011)
Manganese sulphate (kg)		0.07				Manganese sulphate (Majeau-Bettez et al., 2011)
Steel (kg)		0.21	0.40	0.35	0.02	Steel, low-alloyed, hot rolled production

*The amounts are adapted to match the input of materials specific to the composition of the analysed battery cell.

**The output of this process is the production of copper.

***It was assumed that all plastic materials were burned during the pyrometallurgical recycling process.

**Fig. 3:**
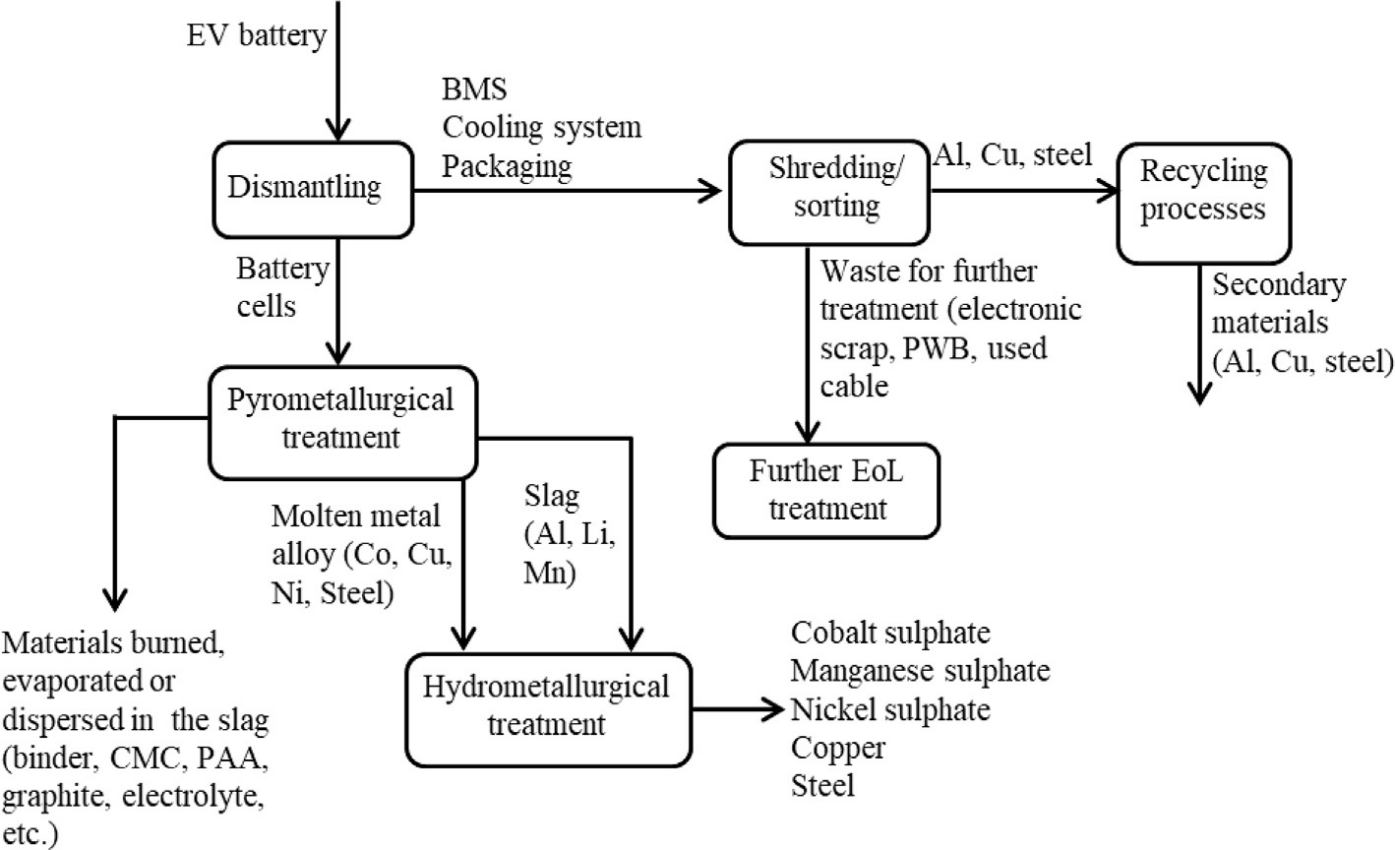
Diagram of the battery components' EoL treatments.

Fig. 3 shows a diagram of the battery components’ EoL treatments.

Although recycling is the current EoL management for retired EV batteries, it is worth mentioning that, according to several literature studies, retired EV Li-ion batteries still have 80% of their initial capacity intact (Bobba et al., 2018a, 2018b; Gohla-Neudecker et al., 2015; Heymans et al., 2014; Richa et al., 2017). Therefore, before recycling, reusing these in less demanding stationary energy storage applications can be considered as a source of both environmental and economic benefits by avoiding the production of new battery packs (Bobba et al., 2018a), as well as reducing the energy imported from the electricity grid (Guarino et al., 2015). However, nowadays Li-ion traction batteries have to be properly collected and recycled; therefore, in accordance with the in-force Waste Batteries Directive (Directive, 2006/66/EC), the reuse of such batteries has not yet been developed in Europe. Consequently, this paper analyses only recycling, since this is the most common and realistic option currently in Europe.

### 3.3 Life cycle impact assessment: results and interpretation

**Fig. 4:**
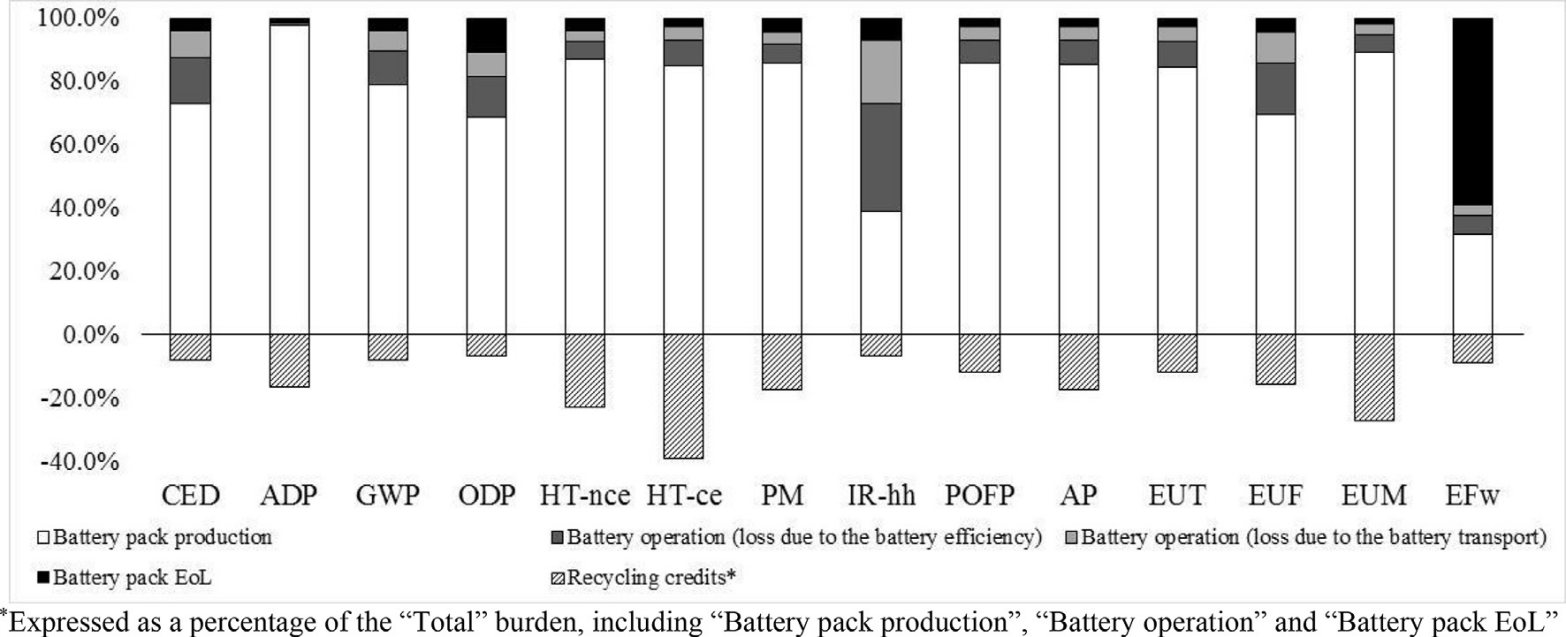
Life cycle environmental impacts – impacts refer to the defined FU (one LMO–NMC battery pack).

The life cycle impact assessment (LCIA) of the FU, calculated using the impact assessment method described in Section 3.1, is illustrated in Table 6. The impacts due to recycling have been separated from the environmental credits arising from avoiding the production of primary materials. The contribution of each life cycle phase is detailed in Fig. 4.

**Table 6:** Life cycle environmental impacts – impacts refer to the defined FU (one LMO–NMC battery pack).

Impact category	Total (without credits)	Recycling credits
CED (MJ)	7.57E+04	−5.85E+03
ADP (kgSb_eq_)	7.75E–02	−1.27E–02
GWP (kgCO_2eq_)	4.52E+03	−3.60E+02
ODP (kgCFC-11_eq_)	3.85E–04	−2.52E–05
HT-nce (CTUh)	2.54E–03	−5.75E–04
HT-ce (CTUh)	4.53E–04	−1.76E–04
PM (kg PM2.5_eq_)	2.92E+00	−5.02E–01
IR-hh (kBqU^235^_eq_)	6.89E+02	−4.49E+01
POFP (kgNMVOC_eq_)	1.32E+01	−1.56E+00
AP (molH^+^_eq_)	3.62E+01	−6.32E+00
EU_T_ (molN_eq_)	4.31E+01	−5.01E+00
EU_F_ (kgP_eq_)	2.67E+00	−4.21E–01
EU_M_ (kgN_eq_)	7.04E+00	−1.90E+00
E_Fw_ (CTUe)	1.93E+05	−1.69E+04

Battery production is the phase mainly responsible for all the impacts considered. In fact, with the exception of the categories ionizing radiation – human health and freshwater eutrophication, the contribution of battery production is always higher than 60%. This trend is consistent with previous LCA studies that have estimated the life cycle impacts of Li-ion traction batteries (Matheys et al., 2009; Schexnayder et al., 2001; Zackrisson et al., 2010). This outcome confirms the importance of understanding the environmental impacts of battery production when assessing the environmental sustainability of electric mobility.

The battery operation phase has a large impact only on ionizing radiation – human health (55%). In the use phase, the impacts of the electricity lost from battery efficiency are about twice those of the electricity lost from battery transport (see Table S2 in the supplementary material).

Battery recycling has a large impact on freshwater ecotoxicity (60%). Besides generating potential environmental impacts, recycling results in environmental credits due to recoverable products, presented as negative values in Table 6 and negative bars in Fig. 4, for the various impact categories (see Section 3.2.3). The environmental credits associated with materials recovered through battery recycling processes exceed the associated environmental impacts linked to the recycling process in all the impact categories examined, with the exception of ozone depletion potential, ionizing radiation and freshwater ecotoxicity. The environmental credits are particularly relevant to the impact categories of marine eutrophication (−27%), human toxicity (about – 20% for human toxicity no cancer effect and −40% for human toxicity cancer effect), particulate matter (−17%) and abiotic resource depletion (−16.4%). This outcome confirms the environmental benefits of recovering Li-ion battery materials, as reported in previous studies (Dewulf et al., 2010; Dunn et al., 2012b; Hendrickson et al., 2015; Richa et al., 2017; U.S. EPA, 2013).

**Fig. 5:**
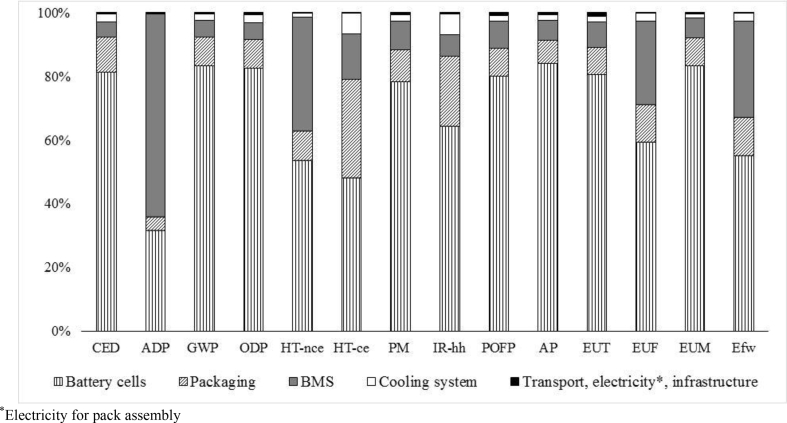
Environmental impacts – battery production phase.

In terms of the production phase, a more in-depth contribution analysis (Fig. 5) shows that battery cell production makes the largest contribution to all of the environmental impact categories examined, with the exception of abiotic depletion potential, in which the largest impacts are attributed to the PWB and cable production for the BMS. This outcome confirms the relevance of cells’ contribution, also reported in several literature studies (Ellingsen et al., 2014; Kim et al., 2016; Majeau-Bettez et al., 2011; Notter et al., 2010).

Battery packaging never exceeds 32%, with the highest contribution made to the human toxicity – cancer effect impact category. For this category, the production of aluminium, steel and copper is responsible for 80% of the overall impact. The production of the cooling system accounts for about 6.5% of the human toxicity – cancer effect and ionizing radiation – human health categories, and contributes less than 3% to all other impact categories. Transport, electricity needed for battery assembly and infrastructure contribute less than 2% to all the categories examined.

The global warming potential of the battery production, calculated per kilowatt hour of battery energy capacity, for ease of comparison with previous studies, is 313 kgCO_2eq_ per kWh of battery energy capacity. This value is in the upper mid-range of estimates found in the literature review summarized in Table 1 and in the study by Ellingsen et al. (2017). It is worth mentioning that the global warming potential per kilowatt hour of battery energy capacity is 190 kgCO_2eq_/kWh (in the mid-range of the literature estimates) if the lower value of the electricity consumption for cell assembly (586 MJ/kWh) among those reported in Ellingsen et al. (2014) is taken instead of the average one and if the average European electricity mix is used instead of the Japanese one. Specifically, the LMO-NMC technology is characterized by a comparable global warming potential with that of the NMC technology, and although it performs worse the LMO one, in this impact category, currently, the interest of the road-transport sector in this chemistry has faded (Zubi et al., 2018). Then, the LMO-NMC technology can be one of the technologies that will contribute to the sustainability of future transport.

According to Ellingsen et al. (2017), large differences in the global warming potential of the production phase can be due to the different energy demands for cell manufacturing and pack assembly. Owing to a dearth of primary data, the greenhouse gas emissions from cell manufacturing are the most difficult aspect of battery production to analyse (Kim et al., 2016). For this reason, energy consumption is a relevant parameter in the sensitivity analysis (Section 6.2.2).

To provide a more reliable comparison with the literature, the authors focused on only the global warming potential of production of the materials of the cell components (i.e. excluding the energy required for cell manufacturing). The literature estimates are between 28 kgCO_2eq_/kWh (for the LMO–NMC battery cell studied by Kim et al. (2016)) and 108 kgCO_2eq_/kWh (for the NMC (LiNi_0.4_Co_0.2_Mn_0.4_O_2_) analysed by Majeau-Bettez et al. (2011)). The value obtained for the LMO–NMC cell analysed in this study is in the mid-range of the literature estimates, at 60 kgCO_2eq_/kWh. Thus, it can be concluded that the LMO–NMC composite cathode technology could represent, besides a good compromise between the higher and the lower energy performance of the NMC and LMO parts, respectively, also a good environmental compromise in terms of global warming potential.

Finally, the cobalt content per kilowatt hour of LMO–NMC cell capacity was estimated and compared with the NMC cell technology presented by Ellingsen et al. (2014). Specifically, the LCI of the NMC cell was extracted, recompiled and implemented in the software used for the LCA study. The results of the analysis show that the LMO–NMC cell technology contains 0.20 kgCo/kWh, while the NMC technology contains 0.560 kg Co/kWh. Therefore, the LMO–NMC cell technology could provide lower costs and less vulnerability to supply disruption than the NMC cell technology, because of the lower cobalt content.

#### 3.3.1 Cell contribution analysis: components

As the cells are responsible for the main energy and environmental contributions, and they are the battery components for which the authors have primary data, the production process of one cell (energy capacity 142.5 Wh; weight 1396.20 g) is examined in detail.

**Fig. 6:**
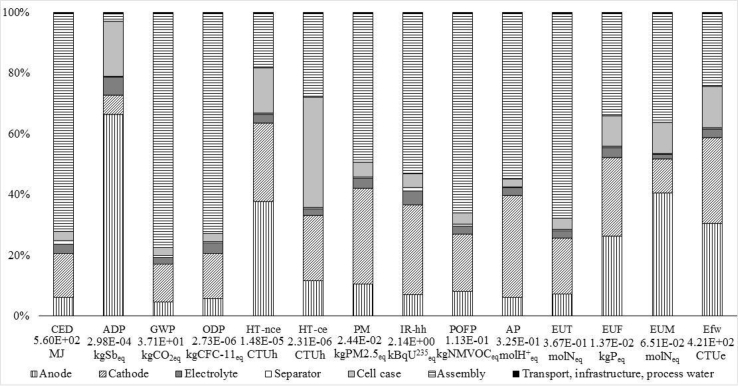
Life cycle impacts of the battery cell production phase.

The LCIA results and the contribution of the different battery cell components are illustrated in Fig. 6.

The analysis of the results obtained shows that in cell production the cell assembly process is responsible for the greatest impacts in almost all of the impact categories investigated. For example, it accounts for about 80% of global warming potential, 70% of ozone depletion potential and cumulative energy demand and around 65% for photochemical ozone formation and terrestrial eutrophication. Therefore, to increase the sustainability of battery production it is necessary to reduce the impacts of the energy consumed during cell assembly by adopting more efficient processes and technologies and by increasing the use of cleaner energy sources (e.g. RESs) (Beccali et al., 2007). The exceptions are abiotic depletion potential, for which the highest contribution (66%) is from anode production, and human toxicity, freshwater and marine eutrophication, and freshwater ecotoxicity, for which the highest impacts (more than 50%) are due to both the anode and cathode production processes. It is worth mentioning that, although cobalt (contained in the cathode) is designated a critical raw material, the greatest impact on abiotic resource depletion is attributable to the anode production process and, specifically, copper primary production, which accounts for 77% of the total impact of cell production on this category. In fact, the criticality of cobalt is mainly attributable to political and economic reasons that are not captured by an environmental impact category. Abiotic depletion potential is one of the few indicators that relates to the consumption of non-energy resources (Ardente et al., 2017). However, its characterization factors do not reflect regional differences in resource consumption, or quality losses during, for example, material use and EoL treatments (Hiete, 2013).

Concerning the criticality of raw materials, several methods are available in the literature for its assessment, like the methods proposed by Graedel et al. (2012) and Nassar et al. (2012) that consider three dimensions of criticality: supply risk, environmental implications, and vulnerability to supply restriction. However, the present article does not aim to perform an assessment of criticality of the metals contained in the battery pack. The authors referred to the 2017 list of Critical Raw Materials for the EU, as identified by the European Commission (European Commission, 2017), and to key policy documents for the battery sector (European Commission, 2018). Based on these documents the authors identified the cobalt as one of the main critical raw material contained in the examined battery cell. In the method applied by the European Commission, the criticality of a raw material is assessed based on its economic importance and supply risk. The European Commission criticality methodology is considered reliable and robust. In fact, it is the result of an intense and active dialogue with multiple stakeholders, it is based on the use of best available data reflecting the current situation and recent past (Blengini et al., 2017a, Blengini et al., 2017b)”.

A detailed contribution analysis of the cell sub – components highlights that the NMP production is the main contributor, after the cell assembly process, to several impact categories examined. For this reason, the NMP has been identified as a potential relevant parameter for the sensitivity analysis.

The detailed analysis of the contribution to the impacts of the cells is illustrated in Fig. S2 in the supplementary material.

#### 3.3.2 Battery pack recycling: contribution analysis

The detailed LCIA results for the recycling of each battery component are provided in Table 7, and the detailed process contribution is illustrated in Figs. S3–S10 in the supplementary material.

**Table 7:** Life cycle impacts of recycling the battery components.

Impact category	Recycling process – cell	Recycling process – BMS	Recycling process – cooling system	Recycling process – battery packaging
CED (MJ)	2.57E+03	4.75E+01	1.16E+02	4.18E+02
ADP (kgSb_eq_)	6.19E–04	1.17E–04	1.20E–05	2.00E–04
GWP (kgCO_2eq_)	1.48E+02	4.41E+00	7.40E+00	2.58E+01
ODP (kgCFC-11_eq_)	3.73E–05	2.31E–07	8.92E–07	2.85E–06
HT-nce (CTUh)	4.82E–05	1.61E–05	3.98E–06	3.30E–05
HT-ce (CTUh)	9.33E–06	6.32E–07	1.96E–07	1.46E–06
PM (kg PM2.5_eq_)	1.20E–01	2.89E–03	2.02E–03	1.01E–02
IR-hh (kBqU^235^_eq_)	4.20E+01	6.39E–01	7.73E–01	4.19E–00
POFP (kgNMVOC_eq_)	2.99E–01	1.03E–02	1.06E–02	4.77E–02
AP (molH^+^_eq_)	8.37E–01	2.60E–02	2.17E–02	1.06E–01
EU_T_ (molN_eq_)	1.05E+00	4.02E–02	3.27E–02	1.62E–01
EU_F_ (kgP_eq_)	9.24E–02	8.14E–03	1.83E–03	1.84E–02
EU_M_ (kgN_eq_)	1.16E–01	3.95E–03	3.30E–03	1.80E–02
E_Fw_ (CTUe)	1.94E+03	2.33E+03	3.08E+04	7.87E+04
Impact category	Recycling credits – cell	Recycling credits – BMS	Recycling credits – cooling system	Recycling credits – packaging
CED (MJ)	−1.23E+03	−1.27E+02	−1.13E+03	−3.35E+03
ADP (kgSb_eq_)	−1.00E–02	−8.90E–04	−6.64E–05	−1.73E–03
GWP (kgCO_2eq_)	−6.90E+01	−8.40E+00	−6.99E+01	−2.13E+02
ODP (kgCFC-11_eq_)	−6.03E–06	−5.94E–07	−4.62E–06	−1.40E–05
HT-nce (CTUh)	−3.85E–04	−2.91E–05	−2.30E–05	−1.38E–04
HT-ce (CTUh)	−4.31E–05	−7.34E–06	−2.47E–05	−1.01E–04
PM (kg PM2.5_eq_)	−2.59E–01	−1.11E–02	−5.31E–02	−1.79E–01
IR-hh (kBqU^235^_eq_)	−1.04E+01	−1.02E+00	−8.81E+00	−2.47E+01
POFP (kgNMVOC_eq_)	−6.64E–01	−3.86E–02	−1.97E–01	−6.55E–01
AP (molH^+^_eq_)	−3.89E+00	−8.45E–02	−5.90E–01	−1.75E+00
EU_T_ (molN_eq_)	−2.04E+00	−1.30E–01	−6.77E–01	−2.17E+00
EU_F_ (kgP_eq_)	−2.30E–01	−1.75E–02	−3.59E–02	−1.38E–01
EU_M_ (kgN_eq_)	−1.36E+00	−1.14E–01	−6.95E–02	−3.60E–01
E_Fw_ (CTUe)	−9.55E+03	−7.05E+02	−1.34E+03	−5.34E+03

Cell recycling using a pyrometallurgical-hydrometallurgical process results in environmental credits in almost all the impact categories examined, with the exceptions of cumulative energy demand, climate change, ozone depletion potential and ionizing radiation. The main contributions to the recycling impacts relate to electricity consumption, sodium hydroxide production and waste treatment, which overall account for more than 58% in all the impact categories examined. Therefore, the benefits of recycling could be increased by reducing its energy intensity and/or using energy generated by RESs. The environmental credits related to cobalt, nickel and manganese sulphates, copper and steel are significant in the abiotic depletion potential, human toxicity – no cancer effect, acidification potential and marine eutrophication impact categories, in which they account for 79%, 67%, 62% and 71%, respectively, of the total environmental credits.

. However, the environmental benefits of recycling could be increased if the other cell components/materials, such as graphite, electrolyte and aluminium, were recovered, i.e. by designing battery cells to make disassembling and separating the cell components easier and more secure (Cellura et al., 2014). Moreover, if reuse in stationary energy storage applications is envisaged, this strategy could be useful to guarantee easy disassembly of modules into cells to test the failure rate of the cells (Ahmadi et al., 2017; Bobba et al., 2018a).

Regarding the other battery components, the LCIA highlights that the greatest contributions to the recycling impacts are associated with energy consumption (heat and electricity) and with preparing copper scraps for recycling. These contribute more than 60% to all the impact categories examined, with the exception of freshwater ecotoxicity, in which the preparation of aluminium scraps for recycling accounts for about 98%. With regard to the environmental credits related to the avoidance of the production of the copper, aluminium and steel recovered from the BMS, cooling system and packaging, these are significant in the cumulative energy demand, global warming potential, ionizing radiation, human toxicity – cancer effect and ozone depletion potential impact categories, in which they account for 79%, 81%, 77%, 76% and 76%, respectively, of the total environmental credits.

The results obtained show that, although the most valuable metals (cobalt, nickel and copper) are contained in the cathode, the recycling of other materials, such as aluminium, copper and steel, contained in the other battery components increases the environmental benefits of battery recycling. Appropriate battery design could make it easier to separate the battery components and thereby optimize the recovery of the various metal fractions.

## 4 Sensitivity analysis

Although the LCA is a useful tool for estimating the effective energy and environmental impacts of a product or service, its reliability strictly depends on complete and precise data, which are not always available (Ardente et al., 2005; Cellura et al., 2011). Because of the lack of primary data from industry, several assumptions have been made in this study. Hence, the authors performed a sensitivity analysis, based on a scenario analysis, to assess the influence of the assumptions on the results obtained. As discussed by Igos et al. (2018), this approach also allows the uncertainty resulting from input data to be embodied and modelled. Compared with the “base case” analysis (as described in previous sections), “worst” and “best” scenarios were set by assuming one-at-a-time parameter change. Relevant parameters for the scenario analysis were identified according to the LCIA outcomes. The values of these parameters for the “worst” and “best” scenarios were decided on using data from the literature. With regard to the production stage, the sensitivity analysis was performed for the battery cells only, as details on the other battery components were inferred from literature studies.

The assessment of battery cell production was based on inputs on materials obtained from disassembly experiments performed on a case-study battery cell, while the energy required for cell assembly was inferred from literature data (Ellingsen et al., 2014). As discussed in Section 3.2.1 and highlighted in Table 4, for the negative active material, the binder, the solvent, the electrolyte and the cell case, assumptions were made about the amount used in the cell and in the specific material composition; moreover, the electricity required for cell assembly was another input to the LCA model affected by high uncertainty. The main assumptions about the cell production process are detailed in Table S3 in the supplementary material.

The LCIA results showed that the negative active material, the binder, the solvent and the cell case were not major contributors to battery cell production, as their incidences were less than 3% in all the impact categories examined; therefore, they were not taken into account in the sensitivity analysis. With regard to the electrolyte, its composition is based on previous studies; however, it was not possible to identify a detailed composition in the literature that differed from that assumed in the present study (including the option of using water, as mentioned by Zackrisson et al. (2010)).

The sensitivity analysis was therefore carried out for two parameters: the amount of solvent and the electricity required for cell assembly.

For the use phase, a sensitivity analysis was performed to assess how the assumptions about electricity mix, battery efficiency and weight–energy relationship influenced the results obtained. For the EoL phase, the goal of the sensitivity analysis was to assess how lower recycling rates (than the values in Table S1 (supplementary material)) considered as the base case could affect the overall life cycle impacts.

The main assumptions of the scenario analysis are shown in Table 8. For each parameter (according to both the LCIA outcomes and the uncertainty at input level) a worst and a best scenario were defined with respect to the base case by using data from the literature or the authors making their own arbitrary variations (Igos et al., 2018). This scenario analysis permitted, in accordance with Igos et al. (2018), to perform a sensitivity analysis including a rough estimation consideration of the uncertainty related to the input data.

**Table 8:** Main assumptions of the scenario analysis.

Life cycle phase	Parameters	Base case	Worst scenario	Best scenario
Production	• NMP	• NMP, 0.4 kg/kg of positive electrode paste; 0.94 kg/kg of negative electrode paste (Ellingsen et al., 2014)	• 0.82 kgNMP/kg for both positive and negative electrode paste (Cerdas et al., 2018).	• 0.28 kgNMP/kg for both positive and negative electrode paste (Majeau-Bettez et al., 2011);
	• Electricity for cell assembly	• 960 MJ/kWh of battery cell capacity (Ellingsen et al., 2014)	• 2318 MJ/kWh of battery capacity (Ellingsen et al., 2014)	• 309 MJ/kWh of battery capacity (Cerdas et al., 2018)
Use	• Electricity mix for battery charging	• European average (own assumption)	• Coal-based mix (Chinese electricity mix) – CN scenario	• RES-based energy mix (e.g. the Norwegian one, mainly based on hydropower) – NO scenario
	• Battery charging efficiency	• 95% (Bobba et al., 2018a)	• 90% (Zackrisson et al., 2010)	• 98% (Bobba et al., 2018a)
	• Weight–energy relationship	• 30% (Zackrisson et al., 2010)	• 50% (higher electricity Consumption for battery transport) (Zackrisson et al., 2010)	• 15% (lower electricity Consumption for battery transport) (Zackrisson et al., 2010)
	• Driven range (km)	• 136,877 (own primary data)	• 96,000 (−30% compared with base case)	• 180,000 (+30% compared with base case
EoL	• Recycling rate	• Table S1 (Chancerel and Marwede, 2016)	• –30%	–

With regard to the production phase, the sensitivity analysis highlights that varying the NMP amount in the range examined does not affect the results obtained significantly, as the percentage variation of the impacts in the worst and best scenarios, if compared with the base case, are lower than +/−5% in all the impact categories examined (see Table S4 in the supplementary material).

In terms of the electricity consumed during cell assembly, in both scenarios (worst and best) battery production remains the life cycle phase responsible for the highest impacts in almost all the categories examined (see Tables S5 and S6 in the supplementary material). The electricity consumption during cell assembly has a large effect on the environmental assessment (see Table S7 in the supplementary materials). The results prove overall the relevance of further investigating this aspect in future studies, possibly using primary data from industry.

With regard to the operation phase, the sensitivity analysis highlights that a different electricity mix has a large effect on the results obtained (see Table S8). In particular, with regard to the impact on global warming potential, an increase of about 25% in the worst scenario and a reduction of about 17% in the best scenario were observed, compared with the base case scenario. In terms of battery efficiency and the weight–energy relationship (see Tables S9 and S10 in the supplementary material), this parameter only slightly affected the results obtained. Moreover, the impacts of electricity losses due to battery efficiency are larger than those caused by the electricity consumed by battery transport in both scenarios examined, although in the worst scenario they become more similar. The impact of battery transport becomes larger than that of battery efficiency when the latter is 98%. Battery efficiency has a large effect on the results obtained (see Tables S11, S12 and S13 in the supplementary material).

Finally, the sensitivity analysis of the driving range shows that the environmental impacts, expressed per kilometre, increase by about 35% (average value) in the worst scenario, while in the best scenario they decrease by about 20% (average value) compared with the base case (see Table S14 in the supplementary material). Therefore, increasing the lifetime of batteries could significantly improve the environmental sustainability of electric mobility.

The sensitivity analysis confirms that adopting a more renewable electricity mix can significantly improve the impacts relates to the battery use phase. Moreover, it confirms that battery efficiency, more than battery weight, is a key factor in reducing the impacts of the battery use phase. Finally, a greater driving range significantly improves the environmental sustainability of electric mobility.

The sensitivity analysis of EoL treatment shows that if recycling rates are reduced by 30% the impact of the recycling processes becomes greater than the environmental credits associated with the recovered products also for the cumulative energy demand and global warming potential impact categories, in addition to ozone depletion potential, ionizing radiation and freshwater ecotoxicity in the base case (see Table S15 in the supplementary material). This proves the importance of ensuring high recovery levels for the various material fractions, for example through appropriate design of batteries’ EoL, and proper dismantling and sorting of the waste battery components for recycling.

## 5 Conclusions

Traction EV batteries are considered the key element for the deeper decarbonisation of the transport sector. Considering the increasing forecasted popularization of EVs, it is vital that we assess the environmental impacts connected with traction EV batteries by adopting a life cycle perspective. In this context, the authors applied the LCA methodology to a Li-ion traction battery pack usable in PHEVs to assess the life cycle stages responsible for the main impacts and the potential mitigation achievable through recycling. The analysis was carried out with reference to one battery pack, for which the authors provide the BoM of the cells, compiled using both primary and literature data.

The data and results of this study allow the expansion of the state of the art in relation to Li-ion traction batteries, providing the first contribution analysis of the materials in an LMO–NMC cell technology for PHEVs and the first assessment of the energy and environmental data related to its production and recycling processes.

The study confirms that the battery production is the phase responsible for the greatest contribution to life cycle impacts. The electricity required for cell assembly is responsible for the main impacts. The amount of electricity required for cell assembly varies widely in the literature examined. However, the sensitivity analysis carried out with reference to this parameter highlights that, even when considering the lowest value available in the literature, battery production remains the stage with the greatest life cycle impact, and cell assembly remains the phase responsible for the greatest contribution to the cumulative energy demand, global warming potential and ozone depletion potential impact categories. Although it would be preferable to increase the reliability of the assessment by using primary data from battery manufacturers, the results obtained nevertheless allow some recommendations for decreasing the impact of EVs to be made, in particular reducing the electricity consumed during battery production and using a low carbon electricity mix, especially considering the target of reducing overall global warming potential. The comparison of the production phase of the LMO–NMC battery cell with that of other cell technologies highlights that the LMO–NMC cell can contribute, with other battery chemistries like NMC, to the sustainability of future transport. In fact, in addition to a good compromise between the higher and lower performances of the NMC and LMO technologies, it is characterized by a comparable global warming potential with the NMC technology that actually dominates in EV and PHEV applications. Moreover, compared with the NMC cell technology, it can result in lower costs and less vulnerability to supply disruption, because of its lower cobalt content.

With regard to the use phase, this accounts, on average, for about 20% of the overall life cycle impact. Moreover, a deeper analysis highlights that the impact of electricity losses due to battery efficiency can be up to 30% greater for certain impact categories than that due to battery transport. This outcome confirms that battery efficiency is a very important parameter for the battery use phase. Impacts on the operation phase due to increased battery mass were generally low.

Moreover, the sensitivity analysis shows that battery production is also the phase with the largest impact in the worst use phase configuration (90% battery efficiency; 50% weight–energy relationship). Finally, the results of the analysis of EoL treatment show that recycling the battery is environmentally beneficial for almost all of the impact categories examined; however, to increase the sustainability of traction battery production it is important to recover not only the valuable materials contained in the cells but also the materials contained in other battery components. With this in mind, battery components could be designed to enable easy and secure separation of the various material fractions and increase the recycling rates of those that are recoverable.

The assessment of a wide range of environmental impact categories allowed to identify the processes that are responsible for the highest contributions for the different environmental impacts considered. An assessment based on a multi-indicator approach can provide a more comprehensive information to battery designers to avoid the potential shifting of the impacts from one impact category to another.

Considering that the production stage accounts for the highest impact in almost all the categories examined, and in all the configurations considered in the sensitivity analysis, an LCA analysis based on primary data provided by the battery industry is urgently required, in particular for the energy required in cell assembly. This would allow a more reliable set of environmental data to be provided to decision makers to improve the design of future batteries. Moreover, this outcome suggests that consideration should be given to extending traction batteries’ lifetime as a further strategy to increase their sustainability beyond the environmental benefits provided by recycling at EoL.

## Disclaimer

The views expressed in the article are personal and do not necessarily reflect an official position of the European Commission. Neither the European Union institutions and bodies nor any person acting on their behalf may be held responsible for the use which may be made of the information contained therein.

## Footnotes

1 Forecast for the Reference Technology Scenario, which reflects projections that respond to energy strategies (energy efficiency, diversification and decarbonisation) that have been announced.

2 PHEV: an EV that has both an ICE and an electric motor with a large battery that can be recharged by plugging it into an external source of electric power.

3 For the modelling of the EoL, the “recyclability substitution” approach has been applied. This assumes that the recycled materials produced at the EoL of the product will retain the properties of the original material input to the life cycle, and credits the product with displacing virgin material production in proportion to the recyclability rate (Allacker et al., 2014).

4 Ellingsen et al. (2014) presented three possible values for electricity consumption for cell manufacturing: 586 MJ/kWh, 960 MJ/kWh and 2318 MJ/kWh. In the present article, the average value (960 MJ/kWh) has been used.
