# Supplementary material for: Energy and environmental assessment of a traction lithium-ion battery pack for plug-in hybrid electric vehicles
Source: J Clean Prod. 2019 Apr 1;215:634–49. doi: 10.1016/j.jclepro.2019.01.056 (PMC6472661; doi:10.1016/j.jclepro.2019.01.056)
Supplement: Multimedia component 1 [file mmc1.docx]

Energy and environmental assessment of a traction lithium-ion battery pack for plug-in hybrid electric vehicles

Maria Anna Cusenza^a^, Silvia Bobba^bc^, Fulvio Ardente^b*^, Maurizio Cellura^a^, Franco Di Persio^d^

^a^University of Palermo, Department of Energy, Information Engineering and Mathematical Models (DEIM), Viale delle Scienze Building 9, Palermo, Italy

^b^European Commission, Joint Research Centre, Directorate for Sustainable Resources, via Enrico Fermi 2749, Ispra, VA, Italy

^c^DIATI – Department of Environment, Land and Infrastructure Engineering, Politecnico di Torino, Corso Duca degli Abruzzi 24, Turin, Italy

^d^European Commission, Joint Research Centre, Directorate for Energy, Transport and Climate, Energy Storage Unit, Westerduinweg 3, NL-1755 LE Petten, the Netherlands

^*^Corresponding author. Email address: Fulvio.ARDENTE@ec.europa.eu

**Supplementary material**

# Description of cell components

The cathode is composed of a positive current collector (usually an aluminium foil) with a coat of positive electrode paste. The positive electrode paste is composed of a positive active material (commonly made of lithium metal oxide material), a binder and carbon black, which improves conductivity. The cathode releases lithium ions to the anode during charging and receives lithium ions during discharging. The anode is composed of the negative current collector (usually a copper foil) with a coat of negative electrode paste that consists of a negative active material (usually graphite) and a small amount of binder. The anode receives lithium ions from the cathode during charging and releases lithium ions during discharging. The electrolyte is the medium through which the charge is transferred in the cell. It usually consists of an expensive 1-molar solution of a lithium salt in an inexpensive organic solvent such as propylene carbonate or dimethyl carbonate (Gaines and Cuenca, 2000), plus some additives (e.g. carbon black to improve conductivity). Usually, the amount of the additive in the electrolyte is no more than 5% either by weight or by volume, although its presence significantly improves the Li-ion battery’s performance (Zhang, 2006). The separator separates the cathode from the anode to prevent short circuits between them. It has a microporous polymer membrane allowing Li ions to pass through the pores.

# Cell disassembly and material breakdown

A new LMO–NMC cell was disassembled in a glove box in an inert argon atmosphere and a material breakdown analysis was performed. During all the disassembling steps, the weights of the detached elements and of the leftover material were recorded to keep track of evaporated electrolyte and any materials lost during dismantling.

Electrolyte recollection can be challenging because of its volatility. A portion of liquid electrolyte is usually free inside the cell because it has not yet been completely absorbed by the active materials. The same behaviour has been observed in cells produced by other manufacturers (Lebedeva et al., 2015). The free electrolyte is first recollected through two holes drilled into the steel metal case. Then the metal case is removed, revealing two packages (jelly rolls) connected in parallel at the positive and negative terminals through a copper and aluminium current collector. Each of the two jelly rolls is made of three layers (cathode, anode and separator) rolled into a prismatic shape and wrapped with a soft plastic cover to keep the active materials soaking in the electrolyte. One package is then opened and unrolled to separate the three layers (see Fig. S1)


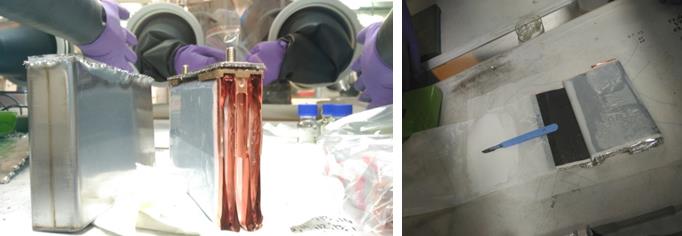


Fig. S1 Left, components of a fresh LMO–NMC/graphite cell after opening and removing the cell casing in a glove box; right, unfolding of one of the two prismatic jelly rolls

The dismantling and subsequent analysis are performed by carrying out a material breakdown, separating steel (external case, connectors and terminals), aluminium and copper (current collectors and electrode foils), polymer (wrapping, separator and tapes), cathode and anode active material, binder (for the anode and the cathode), carbon black (in the cathode) and, finally, electrolyte. One limit of the process lay in the impossibility to discriminate the fractions of binder and carbon black in the cathode composition and fraction of binder in the anode composition. To overcome such challenge an average value for the electrode composition is taken based on data available on literature for NMC and LMO cathodes (Li et al., 2011) and graphite anodes (Argonne National Laboratory, 2011; Liu et al., 2014). Big scatter of the carbon black fraction results in a lower accuracy value. Eventually, assumption on the evaporated electrolyte during the dismantling process is made. These results of the cell material breakdown are shown in Table 3 in the manuscript, where the average weight of all those elements is provided (% in weight and fraction in grams) including error estimation (+/– g). These results are shown in Table 3 in the main paper.

The most difficult estimation was that of the composition of the active material, to distinguish the contributions of the binder and the remaining electrolyte for the anode and the cathode and the carbon black for the cathode. Carbon black resulted in the poorest accuracy due to the scarcity of available data in the literature.

# Inventories for the EoL treatment of the battery cells, the BMS, the packaging and the cooling system

The inventories for the EoL of the battery cells, the BMS, the packaging and the cooling system are detailed in Table S1. The eco-profiles of the materials and energy sources used in battery recycling are based on the Ecoinvent database (Frischknecht et al., 2005; Wernet et al., 2016). The recycling rates of the recoverable materials embedded both in the cells and in other battery components are inferred from Chancerel and Marwede (Chancerel and Marwede, 2016) and are summarized in Table S1.

Table S1 Recoverable materials from battery pack components and corresponding recycling rates

| **Materials** | **Recycling rate (%)** | **Material recovered from the cells** | **Material recovered from other components** |
| --- | --- | --- | --- |
| Aluminium | 98 | NR^*^ | R^**^ |
| Cobalt (as cobalt sulphate) | 90 | R | NP^***^ |
| Copper | 95 | R | R |
| Nickel (as nickel sulphate) | 90 | R | NP |
| Manganese (as manganese sulphate) | 90 | R | NP |
| Steel | 98 | R | R |

^*^NR, not recovered; ^**^R, recovered; ^***^NP, not present

The recycling processes of the BMS, cooling system and battery packaging are modelled taking into account the preparation of the aluminium, iron and copper scraps for recycling and the energy required to recycle each recoverable fraction. PWB, electronic scrap, plastic materials and used cable were considered waste for further treatment. Specifically, for PWB treatment for the recovery of precious metals using a metallurgical process was considered. This included infrastructure for weighting, shredding and sampling the PWB, energy consumption and an estimation of the energy required to transport it. For electronic scrap, the dismantling of the electronic control equipment was considered. Finally, for used cable the treatment process included infrastructure (a shredder, followed by a modern grinding machine with current separation technology), energy consumption and an estimation of the energy required to transport waste cable from electric and electronic devices. For the plastic fraction, an average treatment was considered, such as disposal in landfill, incineration or use as an alternative fuel or as a raw material in clinker production.

# Life cycle impact assessment: results

Table S2 Life cycle environmental impacts – impacts refer to the defined Funtional Unit (one LMO–NMC battery pack)

| **Impact category** | **Battery pack production (%)** | **Battery operation (loss due to battery efficiency) (%)** | **Battery operation (loss due to battery transport) (%)** | **Battery pack EoL (%)** | **Total^+^** | **Recycling credits (%)^*^** |
| --- | --- | --- | --- | --- | --- | --- |
| CED (MJ) | 72.8 | 14.5 | 8.6 | 4.2 | 7.57E+04 | –7.7 |
| ADP (kgSb_eq_) | 97.6 | 0.7 | 0.4 | 1.2 | 7.75E–02 | –16.4 |
| GWP (kgCO_2eq_) | 78.8 | 10.5 | 6.4 | 4.1 | 4.52E+03 | –8.0 |
| ODP (kgCFC-11_eq_) | 68.9 | 12.8 | 7.6 | 10.7 | 3.85E–04 | –6.6 |
| HT-nce (CTUh) | 87.0 | 5.7 | 3.4 | 4.0 | 2.54E–03 | –22.6 |
| HT-ce (CTUh) | 85.0 | 7.8 | 4.6 | 2.6 | 4.53E–04 | –39.0 |
| PM (kg PM2.5_eq_) | 85.6 | 6.1 | 3.6 | 4.6 | 2.92E+00 | –17.2 |
| IR-hh (kBqU^235^_eq_) | 38.8 | 34.1 | 20.2 | 6.9 | 6.89E+02 | –6.5 |
| POFP (kgNMVOC_eq_) | 85.6 | 7.3 | 4.3 | 2.8 | 1.32E+01 | –11.8 |
| AP (molH^+^_eq_) | 85.5 | 7.4 | 4.4 | 2.7 | 3.62E+01 | –17.4 |
| EU_T_ (molN_eq_) | 84.6 | 7.8 | 4.6 | 3.0 | 4.31E+01 | –11.6 |
| EU_F_ (kgP_eq_) | 69.5 | 16.3 | 9.7 | 4.5 | 2.67E+00 | –15.8 |
| EU_M_ (kgN_eq_) | 89.0 | 5.7 | 3.4 | 2.0 | 7.04E+00 | –27.0 |
| E_Fw_ (CTUe) | 31.7 | 6.0 | 3.6 | 58.8 | 1.93E+05 | –8.8 |

^+^‘Battery pack production (%)’ + ‘Battery operation (loss due to battery efficiency) (%)’ + ‘Battery operation (loss due to battery transport) (%)’ + ‘Battery pack EoL (%)’ = 100%

^*^Expressed as a percentage of the “Total” burden, including “Battery pack production”, “Battery operation” and “Battery pack EoL”

# Cell contribution analysis: components


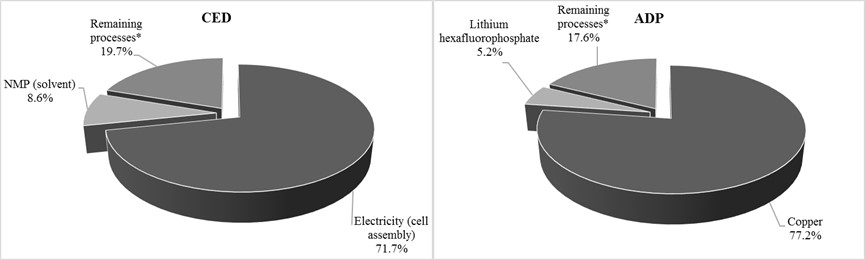


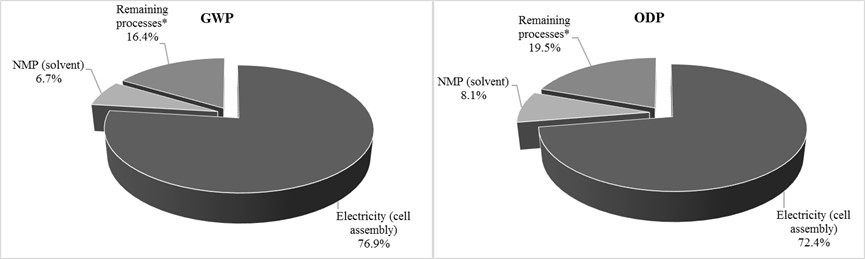

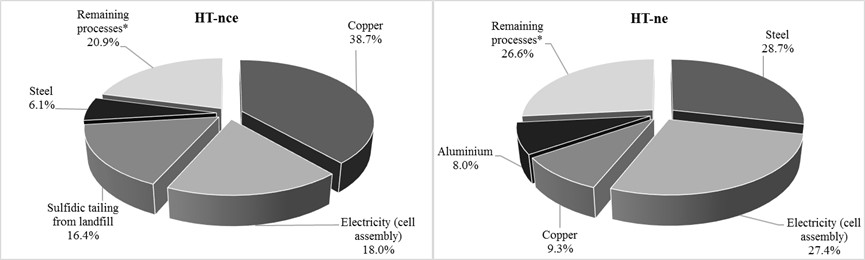

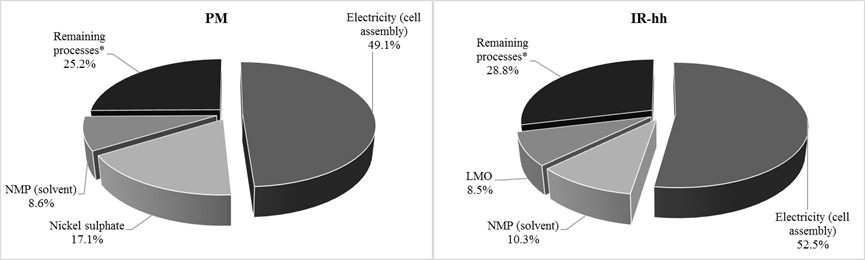

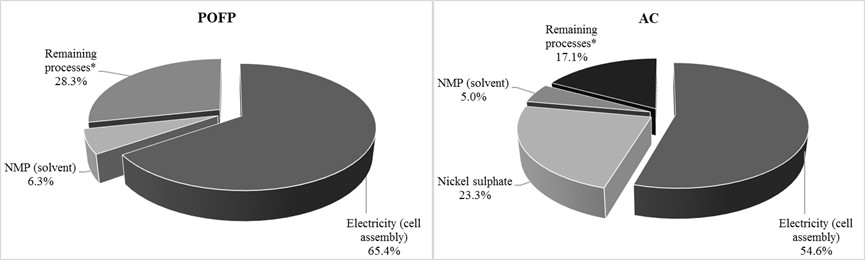


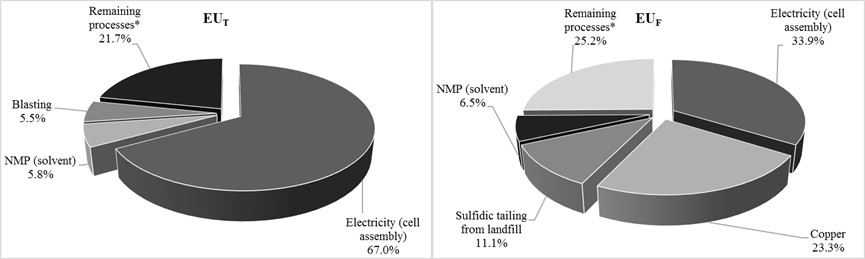

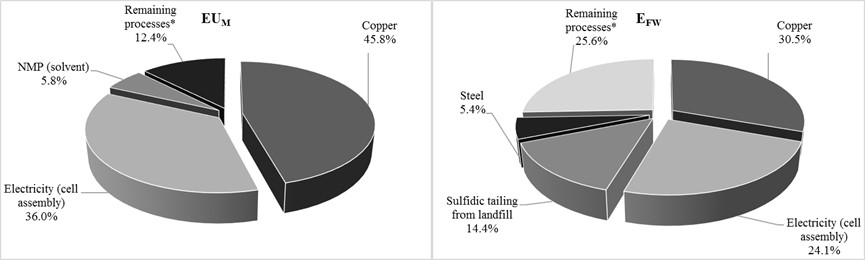


^*^Remaining processes: sum of the processes with a percentage contribution lower than 5%

Fig. S2. Impact related to battery cell production process – process contribution

# Life cycle impacts of recycling the battery components


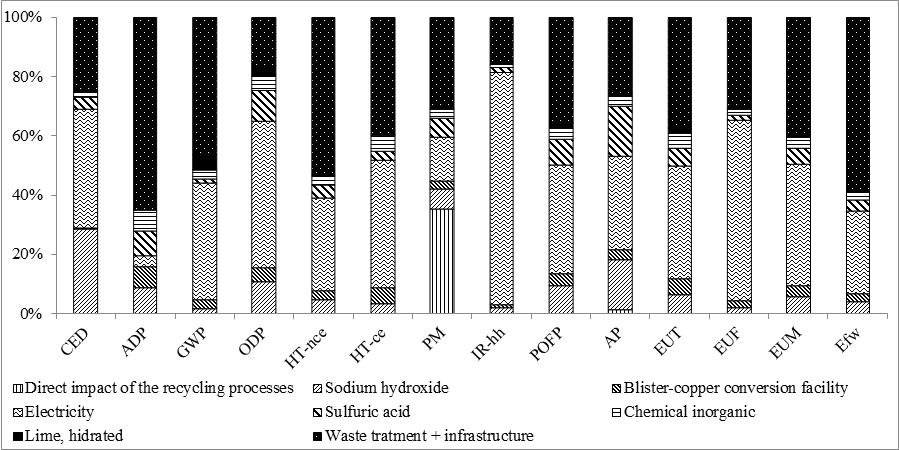


Fig. S3 Impact related to battery cell recycling treatment – process contribution


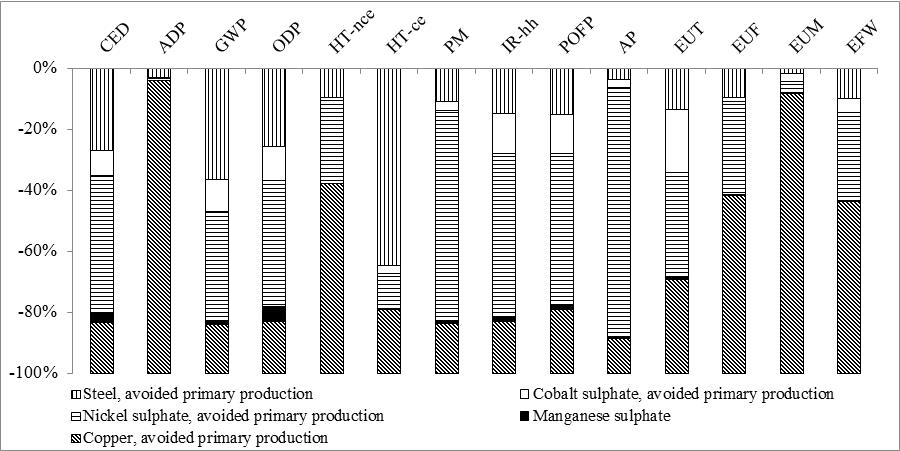


Fig. S4 Environmental credits related to battery cell recycling treatment – process contribution


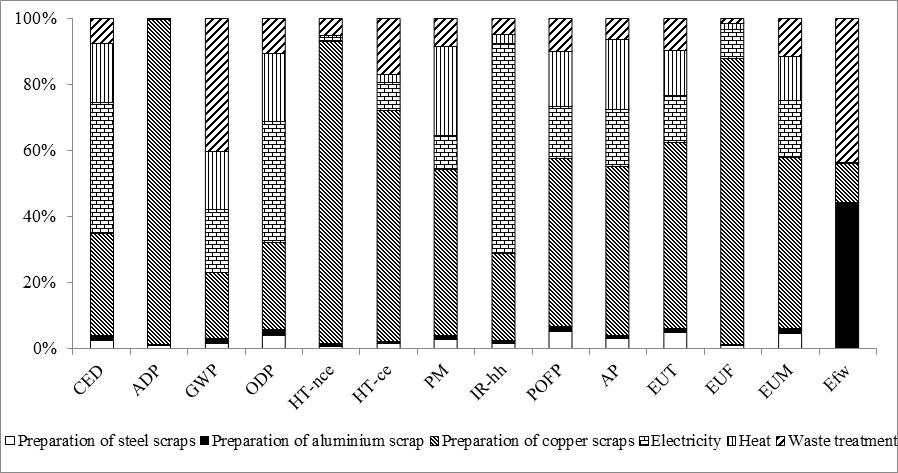


Fig. S5 Impact related to BMS recycling treatment – process contribution


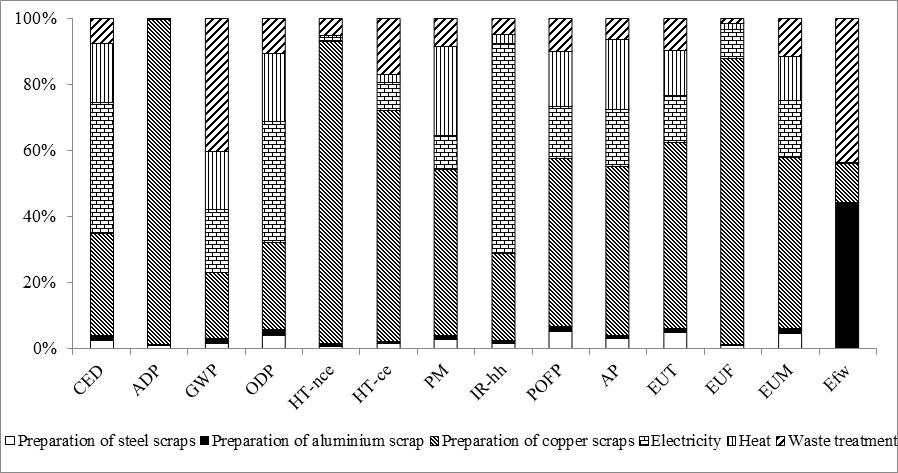


Fig. S6 Environmental credits related to BMS recycling treatment – process contribution


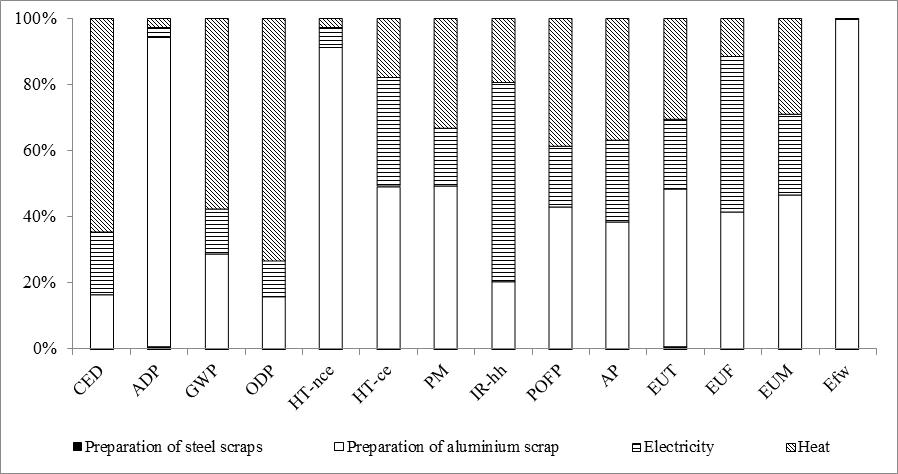


Fig. S7 Impact related to cooling system recycling treatment – process contribution


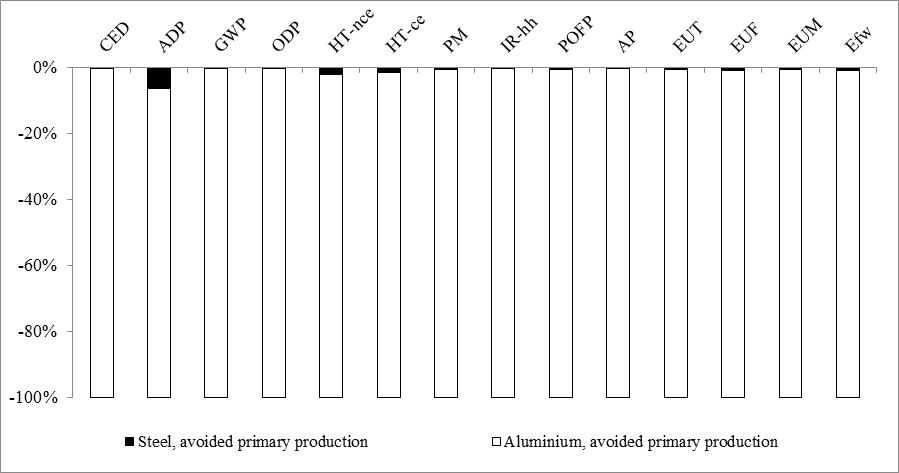


Fig. S8 Environmental credits related to cooling system recycling treatment – process contribution


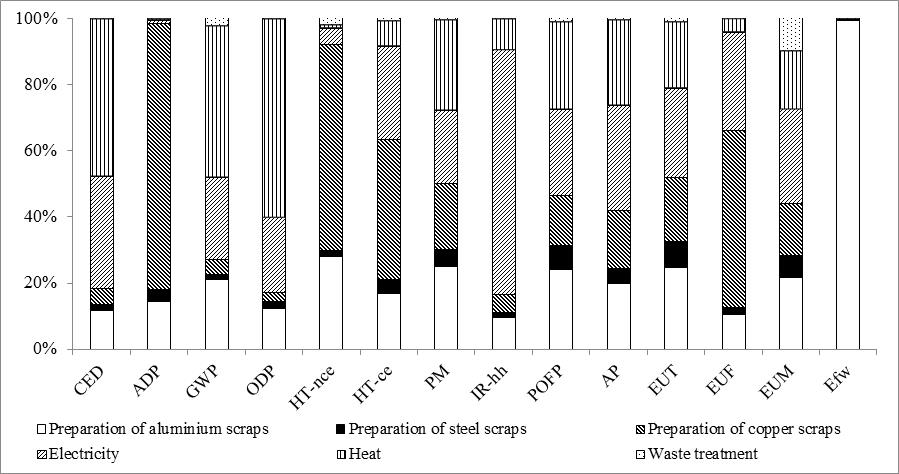


Fig. S9 Impact related to packaging recycling treatment – process contribution


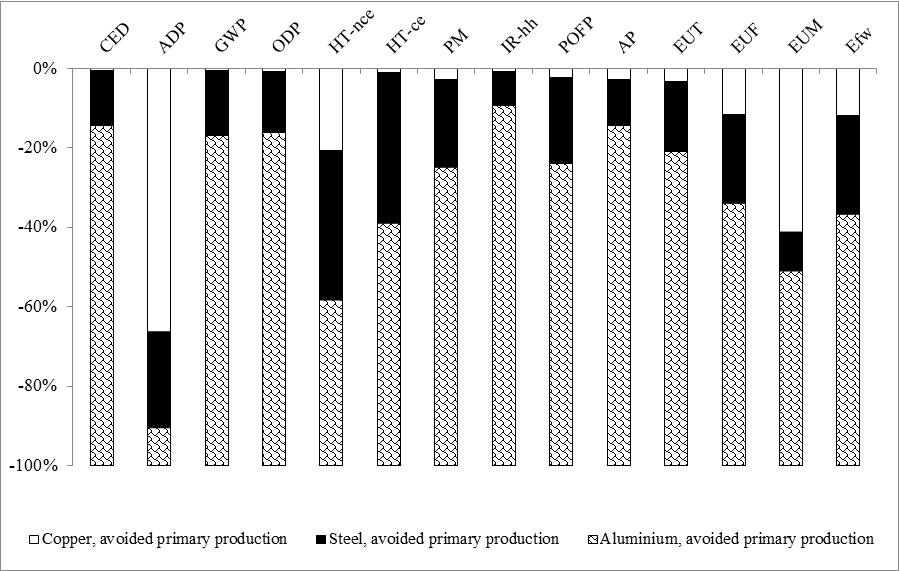


Fig. S10 Environmental credits related to packaging recycling treatment – process contribution

# Sensitivity analysis

## Main assumptions related to the cell production process

Table S3 Main assumptions related to the cell production process

| **Cell component/energy requirement** | **Assumptions for the LCA** |
| --- | --- |
| Negative active material | Material composition: synthetic graphite |
| Binder | Material composition: in the cathode, PAA+CMC; in the anode, PVDF |
| Solvent | Material composition and weight: NMP, 0.4 kg/kg of positive electrode paste; 0.94 kg/kg of negative electrode paste |
| Electrolyte | Material composition: LiPF_6_ + C_3_H_4_O_3_ |
| Separator | Material composition: PP granulate + PE granulate |
| Cell case | Material composition of plastic fraction: PP granulate + PE granulate |
| Cell assembly | Amount: 960 MJ/kWh of battery cell capacity |

## Sensitivity analysis: results

### NMP analysis

Table S4 Sensitivity analysis results – NMP, percentage variations between the scenarios examined and the base case

| **Impact category** | **Base case** | **Worst scenario (%)** | **Best scenario (%)** |
| --- | --- | --- | --- |
| CED (MJ) | 5.51E+04 | 3.4 | –3.4 |
| ARD (kgSb_eq_) | 7.57E-02 | 0.4 | –0.4 |
| GWP (kgCO_2eq_) | 3.56E+03 | 2.7 | –2.7 |
| ODP (kgCFC-11_eq_) | 2.65E-04 | 3.3 | –3.3 |
| HT-nce (CTUh) | 2.21E-03 | 1.0 | –1.0 |
| HT-ce (CTUh) | 3.85E-04 | 1.0 | –1.0 |
| PM (kg PM2.5_eq_) | 2.50E+00 | 3.3 | –3.3 |
| IR-hh (kBqU^235^_eq_) | 2.67E+02 | 3.2 | –3.3 |
| POFP (kgNMVOC_eq_) | 1.13E+01 | 2.4 | –2.5 |
| AP (molH^+^_eq_) | 3.10E+01 | 2.0 | –2.1 |
| EU_T_ (molN_eq_) | 3.65E+01 | 2.3 | –2.3 |
| EU_F_ (kgP_eq_) | 1.85E+00 | 1.9 | –1.9 |
| EU_M_ (kgN_eq_) | 6.26E+00 | 2.3 | –2.4 |
| E_Fw_ (CTUe) | 6.12E+04 | 1.2 | –1.2 |

### Cell assembly analysis

Table S5 Sensitivity analysis results – cell assembly (worst scenario)

| **Impact category** | **Battery pack production (%)** | **Battery operation (%)** | **Battery pack EoL (%)** | **Total (without credits)** | **Recycling credits**^*^ (%) |
| --- | --- | --- | --- | --- | --- |
| CED (MJ) | 82.7 | 14.6 | 2.6 | 1.19E+05 | –4.9 |
| ADP (kgSb_eq_) | 97.6 | 1.2 | 1.2 | 7.83E–02 | –16.2 |
| GWP (kgCO_2eq_) | 87.4 | 10.1 | 2.4 | 7.63E+03 | –4.7 |
| ODP (kgCFC-11_eq_) | 80.0 | 13.1 | 6.9 | 6.00E–04 | –4.2 |
| HT-nce (CTUh) | 88.3 | 8.1 | 3.6 | 2.83E–03 | –20.3 |
| HT-ce (CTUh) | 86.9 | 10.8 | 2.2 | 5.22E–04 | –33.8 |
| PM (kg PM2.5_eq_) | 90.1 | 6.7 | 3.2 | 4.22E+00 | –11.9 |
| IR-hh (kBqU^235^_eq_) | 48.0 | 46.1 | 5.9 | 8.11E+02 | –5.5 |
| POFP (kgNMVOC_eq_) | 91.31 | 7.2 | 1.7 | 2.13E+01 | –7.3 |
| AP (molH^+^_eq_) | 90.6 | 7.7 | 1.8 | 5.55E+01 | –11.4 |
| EU_T_ (molN_eq_) | 90.5 | 7.7 | 1.8 | 6.99E+01 | –7.2 |
| EU_F_ (kgP_eq_) | 74.3 | 21.9 | 3.8 | 3.18E+00 | –13.3 |
| EU_M_ (kgN_eq_) | 91.9 | 6.6 | 1.5 | 9.58E+00 | –19.9 |
| E_Fw_ (CTUe) | 35.4 | 9.0 | 55.6 | 2.04E+05 | –8.3 |

^*^Expressed as a percentage of the “Total” burden, including “Battery pack production”, “Battery operation” and “Battery pack EoL”

Table S6 Sensitivity analysis results – cell assembly (best scenario)

| **Impact category** | **Battery pack production (%)** | **Battery operation (%)** | **Battery pack EoL (%)** | **Total (without credits)** | **Recycling credits^*^ (%)** |
| --- | --- | --- | --- | --- | --- |
| CED (MJ) | 61.8 | 32.4 | 5.9 | 5.39E+04 | –10.8 |
| ADP (kgSb_eq_) | 97.6 | 1.2 | 1.2 | 7.71E–02 | –16.5 |
| GWP (kgCO_2eq_) | 67.7 | 26.0 | 6.2 | 2.97E+03 | –12.1 |
| ODP (kgCFC-11_eq_) | 56.8 | 28.3 | 14.9 | 2.77E–04 | –9.1 |
| HT-nce (CTUh) | 86.2 | 9.6 | 4.2 | 2.40E–03 | –24.0 |
| HT-ce (CTUh) | 83.7 | 13.5 | 2.8 | 4.19E–04 | –42.2 |
| PM (kg PM2.5_eq_) | 81.5 | 12.6 | 5.9 | 2.27E+00 | –22.1 |
| IR-hh (kBqU^235^_eq_) | 32.8 | 59.6 | 7.6 | 6.27E+02 | –7.1 |
| POFP (kgNMVOC_eq_) | 79.4 | 16.6 | 4.0 | 9.20E+00 | –16.9 |
| AP (molH^+^_eq_) | 80.3 | 16.0 | 3.7 | 2.66E+01 | –23.8 |
| EU_T_ (molN_eq_) | 77.7 | 18.0 | 4.3 | 2.98E+01 | –16.8 |
| EU_F_ (kgP_eq_) | 66.3 | 28.7 | 5.0 | 2.42E+00 | –17.4 |
| EU_M_ (kgN_eq_) | 86.5 | 11.0 | 2.5 | 5.76E+00 | –33.0 |
| E_Fw_ (CTUe) | 29.7 | 9.8 | 60.5 | 1.88E+05 | –9.0 |

^*^Expressed as a percentage of the “Total” burden, including “Battery pack production”, “Battery operation” and “Battery pack EoL”

Table S7 Sensitivity analysis results – cell assembly, percentage variations between the scenarios examined and the base case

| **Impact category** | **Base case** | **Worst scenario (%)** | **Best scenario (%)** |
| --- | --- | --- | --- |
| CED (MJ) | 6.99E+04 | 62.4 | –31.2 |
| ADP (kgSb_eq_) | 6.48E–02 | 1.2 | –0.6 |
| GWP (kgCO_2eq_) | 4.16E+03 | 74.5 | –37.3 |
| ODP (kgCFC-11_eq_) | 3.60E–04 | 59.8 | –29.9 |
| HT-nce (CTUh) | 1.97E–03 | 14.7 | –7.4 |
| HT-ce (CTUh) | 2.77E–04 | 24.9 | –12.5 |
| PM (kg PM2.5_eq_) | 2.42E+00 | 53.9 | –27.0 |
| IR-hh (kBqU^235^_eq_) | 6.44E+02 | 19.0 | –9.5 |
| POFP (kgNMVOC_eq_) | 1.17E+01 | 69.0 | –34.5 |
| AP (molH^+^_eq_) | 2.99E+01 | 64.5 | –32.2 |
| EU_T_ (molN_eq_) | 3.81E+01 | 70.1 | –35.0 |
| EU_F_ (kgP_eq_) | 2.25E+00 | 22.5 | –-11.3 |
| EU_M_ (kgN_eq_) | 5.13E+00 | 49.6 | –24.8 |
| E_Fw_ (CTUe) | 1.76E+05 | 6.3 | -3.1 |

### Electricity mix analysis

Table S8 Sensitivity analysis results – electricity mix, percentage variations between the scenarios examined and the base case

| **Impact category** | **Base case** | **Worst scenario (%)** | **Best scenario (%)** |
| --- | --- | --- | --- |
| CED (MJ) | 6.99E+04 | 1.5 | –14.4 |
| ADP (kgSb_eq_) | 6.48E–02 | –0.2 | –0.3 |
| GWP (kgCO_2eq_) | 4.16E+03 | 26.1 | –17.2 |
| ODP (kgCFC-11_eq_) | 3.60E–04 | –19.4 | –20.3 |
| HT-nce (CTUh) | 1.97E–03 | –2.5 | –8.8 |
| HT-ce (CTUh) | 2.77E–04 | –7.4 | –16.7 |
| PM (kg PM2.5_eq_) | 2.42E+00 | 75.9 | –10.4 |
| IR-hh (kBqU^235^_eq_) | 6.44E+02 | –53.2 | –55.3 |
| POFP (kgNMVOC_eq_) | 1.17E+01 | 31.4 | –12.0 |
| AP (molH^+^_eq_) | 2.99E+01 | 23.8 | –13.2 |
| EU_T_ (molN_eq_) | 3.81E+01 | 36.8 | –12.9 |
| EU_F_ (kgP_eq_) | 2.25E+00 | –18.7 | –29.3 |
| EU_M_ (kgN_eq_) | 5.13E+00 | 23.1 | –11.5 |
| E_Fw_ (CTUe) | 1.76E+05 | -1.4 | –2.4 |

### Weight–energy relationship analysis

Table S9 Sensitivity analysis results – weight–energy relationship (worst scenario)

| **Impact category** | **Battery pack production (%)** | **Battery operation (loss due to battery efficiency) (%)** | **Battery operation (loss due to battery transport) (%)** | **Battery pack EoL (%)** | **Total** | **Recycling credits^*^ (%)** |
| --- | --- | --- | --- | --- | --- | --- |
| CED (MJ) | 68.9 | 13.7 | 13.5 | 3.9 | 8.01E+04 | –7.3 |
| ADP (kgSb_eq_) | 97.3 | 0.7 | 0.7 | 1.2 | 7.78E–02 | –16.4 |
| GWP (kgCO_2eq_) | 75.6 | 10.3 | 10.2 | 3.9 | 4.71E+03 | –7.6 |
| ODP (kgCFC-11_eq_) | 65.5 | 12.2 | 12.1 | 10.2 | 4.04E–04 | –6.2 |
| HT-nce (CTUh) | 85.1 | 5.5 | 5.5 | 3.9 | 2.60E–03 | –22.1 |
| HT-ce (CTUh) | 82.4 | 7.6 | 7.5 | 2.5 | 4.67E–04 | –37.8 |
| PM (kg PM2.5_eq_) | 83.6 | 6.0 | 5.9 | 4.5 | 2.99E+00 | –16.8 |
| IR-hh (kBqU^235^_eq_) | 34.2 | 30.0 | 29.7 | 6.1 | 7.81E+02 | –5.7 |
| POFP (kgNMVOC_eq_) | 83.3 | 7.1 | 7.0 | 2.7 | 1.36E+01 | –11.4 |
| AP (molH^+^_eq_) | 83.1 | 7.2 | 7.1 | 2.7 | 3.73E+01 | –16.9 |
| EU_T_ (molN_eq_) | 82.1 | 7.6 | 7.5 | 2.9 | 4.45E+01 | –11.3 |
| EU_F_ (kgP_eq_) | 65.3 | 15.3 | 15.2 | 4.3 | 2.84E+00 | –14.8 |
| EU_M_ (kgN_eq_) | 87.0 | 5.5 | 5.5 | 2.0 | 7.19E+00 | –26.5 |
| E_Fw_ (CTUe) | 30.9 | 5.9 | 5.8 | 57.4 | 1.98E+05 | –8.6 |

^*^Expressed as a percentage of the “Total” burden, including “Battery pack production”, “Battery operation” and “Battery pack EoL”

Table S10 Sensitivity analysis results – weight–energy relationship (best scenario)

| **Impact category** | **Battery pack production (%)** | **Battery operation (loss due to battery efficiency) (%)** | **Battery operation (loss due to battery transport) (%)** | **Battery pack EoL (%)** | **Total** | **Recycling credits^*^ (%)** |
| --- | --- | --- | --- | --- | --- | --- |
| CED (MJ) | 76.1 | 15.1 | 4.5 | 4.4 | 7.25E+04 | –8.1 |
| ADP (kgSb_eq_) | 97.8 | 0.7 | 0.2 | 1.2 | 7.74E–02 | –16.4 |
| GWP (kgCO_2eq_) | 81.4 | 11.1 | 3.3 | 4.2 | 4.38E+03 | –8.2 |
| ODP (kgCFC-11_eq_) | 71.6 | 13.3 | 4.0 | 11.1 | 3.70E–04 | –6.8 |
| HT-nce (CTUh) | 88.5 | 5.8 | 1.7 | 4.1 | 2.50E–03 | –23.0 |
| HT-ce (CTUh) | 87.0 | 8.0 | 2.4 | 2.6 | 4.42E–04 | –39.9 |
| PM (kg PM2.5_eq_) | 87.2 | 6.2 | 1.9 | 4.7 | 2.87E+00 | –17.5 |
| IR-hh (kBqU^235^_eq_) | 43.2 | 37.9 | 11.3 | 7.7 | 6.19E+02 | –7.2 |
| POFP (kgNMVOC_eq_) | 87.5 | 7.4 | 2.2 | 2.8 | 1.29E+01 | –12.0 |
| AP (molH^+^_eq_) | 87.4 | 7.5 | 2.2 | 2.8 | 3.54E+01 | –17.8 |
| EU_T_ (molN_eq_) | 86.6 | 8.0 | 2.4 | 3.1 | 4.21E+01 | –11.9 |
| EU_F_ (kgP_eq_) | 73.0 | 17.1 | 5.1 | 4.8 | 2.54E+00 | –16.6 |
| EU_M_ (kgN_eq_) | 90.5 | 5.7 | 1.7 | 2.0 | 6.92E+00 | –27.5 |
| E_Fw_ (CTUe) | 32.2 | 6.1 | 1.8 | 59.8 | 1.90E+05 | –8.9 |

^*^Expressed as a percentage of the “Total” burden, including “Battery pack production”, “Battery operation” and “Battery pack EoL”

### Battery efficiency analysis

Table S11 Sensitivity analysis results – battery efficiency (worst scenario)

| **Impact category** | **Battery pack production (%)** | **Battery operation (loss due to battery efficiency) (%)** | **Battery operation (loss due to battery transport) (%)** | **Battery pack EoL (%)** | **Total (without credits)** | **Recycling credits^*^ (%)** |
| --- | --- | --- | --- | --- | --- | --- |
| CED (MJ) | 63.3 | 25.1 | 7.9 | 3.6 | 8.70E+04 | –6.7 |
| ADP (kgSb_eq_) | 96.9 | 1.5 | 0.5 | 1.2 | 7.81E–02 | –16.3 |
| GWP (kgCO_2eq_) | 70.9 | 19.3 | 6.1 | 3.7 | 5.02E+03 | –7.2 |
| ODP (kgCFC-11_eq_) | 60.8 | 22.6 | 7.1 | 9.5 | 4.36E–04 | –5.8 |
| HT-nce (CTUh) | 82.2 | 10.7 | 3.4 | 3.8 | 2.69E–03 | –21.4 |
| HT-ce (CTUh) | 78.6 | 14.5 | 4.5 | 2.4 | 4.90E–04 | –36.0 |
| PM (kg PM2.5_eq_) | 80.5 | 11.5 | 3.6 | 4.3 | 3.10E+00 | –16.2 |
| IR-hh (kBqU^235^_eq_) | 28.7 | 50.4 | 15.8 | 5.1 | 9.30E+02 | –4.8 |
| POFP (kgNMVOC_eq_) | 79.7 | 13.5 | 4.2 | 2.6 | 1.42E+01 | –10.9 |
| AP (molH^+^_eq_) | 79.5 | 13.7 | 4.3 | 2.5 | 3.90E+01 | –16.2 |
| EU_T_ (molN_eq_) | 78.3 | 14.4 | 4.5 | 2.8 | 4.66E+01 | –10.8 |
| EU_F_ (kgP_eq_) | 59.5 | 27.9 | 8.8 | 3.9 | 3.12E+00 | –13.5 |
| EU_M_ (kgN_eq_) | 84.1 | 10.7 | 3.3 | 1.9 | 7.45E+00 | –25.6 |
| E_Fw_ (CTUe) | 29.8 | 11.3 | 3.5 | 55.4 | 2.05E+05 | –8.2 |

^*^Expressed as a percentage of the “Total” burden, including “Battery pack production”, “Battery operation” and “Battery pack EoL”

Table S12 Sensitivity analysis results – battery efficiency (best scenario)

| **Impact category** | **Battery pack production (%)** | **Battery operation (loss due to battery efficiency) (%)** | **Battery operation (loss due to battery transport) (%)** | **Battery pack EoL (%)** | **Total (without credits)** | **Recycling credits^*^ (%)** |
| --- | --- | --- | --- | --- | --- | --- |
| CED (MJ) | 79.9 | 6.3 | 9.1 | 4.6 | 6.89E+04 | –8.5 |
| ADP (kgSb_eq_) | 98.1 | 0.3 | 0.4 | 1.2 | 7.72E–02 | –16.5 |
| GWP (kgCO_2eq_) | 84.4 | 4.6 | 6.6 | 4.4 | 4.22E+03 | –8.5 |
| ODP (kgCFC-11_eq_) | 74.8 | 5.6 | 8.0 | 11.6 | 3.54E–04 | –7.1 |
| HT-nce (CTUh) | 90.2 | 2.3 | 3.4 | 4.1 | 2.45E–03 | –23.4 |
| HT-ce (CTUh) | 89.3 | 3.3 | 4.7 | 2.7 | 4.31E–04 | –40.9 |
| PM (kg PM2.5_eq_) | 89.0 | 2.5 | 3.7 | 4.8 | 2.81E+00 | –17.9 |
| IR-hh (kBqU^235^_eq_) | 49.1 | 17.2 | 24.8 | 8.8 | 5.43E+02 | –8.3 |
| POFP (kgNMVOC_eq_) | 89.7 | 3.0 | 4.4 | 2.9 | 1.26E+01 | –12.3 |
| AP (molH^+^_eq_) | 89.6 | 3.1 | 4.4 | 2.9 | 3.46E+01 | –18.3 |
| EU_T_ (molN_eq_) | 88.9 | 3.3 | 4.7 | 3.1 | 4.11E+01 | –12.2 |
| EU_F_ (kgP_eq_) | 77.3 | 7.2 | 10.4 | 5.0 | 2.40E+00 | –17.6 |
| EU_M_ (kgN_eq_) | 92.2 | 2.3 | 3.4 | 2.1 | 6.79E+00 | –28.0 |
| E_Fw_ (CTUe) | 32.9 | 2.5 | 3.6 | 61.0 | 1.86E+05 | –9.1 |

^*^Expressed as a percentage of the “Total” burden, including “Battery pack production”, “Battery operation” and “Battery pack EoL”

Table S13 Sensitivity analysis results – battery efficiency, percentage variations between the scenarios examined and the base case

| **Impact category** | **Base case** | **Worst scenario (%)** | **Best scenario (%)** |
| --- | --- | --- | --- |
| CED (MJ) | 6.99E+04 | 16.2 | –9.7 |
| ADP (kgSb_eq_) | 6.48E–02 | 0.9 | –0.5 |
| GWP (kgCO_2eq_) | 4.16E+03 | 12.0 | –7.2 |
| ODP (kgCFC-11_eq_) | 3.60E–04 | 14.1 | –8.5 |
| HT-nce (CTUh) | 1.97E–03 | 7.5 | –4.5 |
| HT-ce (CTUh) | 2.77E–04 | 13.2 | –7.9 |
| PM (kg PM2.5_eq_) | 2.42E+00 | 7.6 | –4.6 |
| IR-hh (kBqU^235^_eq_) | 6.44E+02 | 37.6 | –22.5 |
| POFP (kgNMVOC_eq_) | 1.17E+01 | 85 | –5.1 |
| AP (molH^+^_eq_) | 2.99E+01 | 9.2 | –5.5 |
| EU_T_ (molN_eq_) | 3.81E+01 | 9.1 | –5.5 |
| EU_F_ (kgP_eq_) | 2.25E+00 | 20.0 | –12.0 |
| EU_M_ (kgN_eq_) | 5.13E+00 | 8.0 | –4.8 |
| E_Fw_ (CTUe) | 1.76E+05 | 6.8 | –4.1 |

### Driving range analysis

Table S14 Sensitivity analysis results – battery efficiency, percentage variations between the scenarios examined and the base case

| **Impact category** | **Base case** | **Worst scenario (%)** | **Best scenario (%)** |
| --- | --- | --- | --- |
| CED (MJ/km) | 5.10E–01 | 31.9 | –18.0 |
| ADP (kgSb_eq_/km) | 4.73E–07 | 42.0 | –23.6 |
| GWP (kgCO_2eq_/km) | 3.04E–02 | 34.7 | –19.5 |
| ODP (kgCFC-11_eq_/km) | 2.63E–09 | 33.3 | –18.7 |
| HT-nce (CTUh/km) | 1.44E–08 | 37.6 | –21.2 |
| HT-ce (CTUh/km) | 2.02E–09 | 33.9 | –19.1 |
| PM (kg PM2.5_eq_/km) | 1.77E–05 | 37.6 | –21.1 |
| IR-hh (kBqU^235^_eq_/km) | 4.70E–03 | 17.8 | –10.1 |
| POFP (kgNMVOC_eq_) | 8.52E–05 | 37.0 | –20.8 |
| AP (molH^+^_eq_/km) | 2.18E–04 | 36.5 | –20.6 |
| EU_T_ (molN_eq_/km) | 2.79E–04 | 36.6 | –20.6 |
| EU_F_ (kgP_eq_/km) | 1.64E–05 | 29.4 | –16.6 |
| EU_M_ (kgN_eq_/km) | 3.75E–05 | 37.3 | –21.0 |
| E_Fw_ (CTUe/km) | 1.29E+00 | 38.1 | –21.5 |

### EoL recycling analysis

Table S15 Sensitivity analysis results – recycling rates in EoL treatments

| **Impact category** | **Total (without credits)** | **Recycling credits** |
| --- | --- | --- |
| CED (MJ) | 7.57E+04 | –4.11E+03 |
| ADP (kgSb_eq_) | 7.75E–02 | –8.97E–03 |
| GWP (kgCO_2eq_) | 4.52E+03 | –2.54E+02 |
| ODP (kgCFC-11_eq_) | 3.85E–04 | –1.77E–05 |
| HT-nce (CTUh) | 2.54E–03 | –4.05E–04 |
| HT-ce (CTUh) | 4.53E–04 | –1.24E–04 |
| PM (kg PM2.5_eq_) | 2.92E+00 | –3.53E+00 |
| IR-hh (kBqU^235^_eq_) | 6.89E+02 | –3.16E+01 |
| POFP (kgNMVOC_eq_) | 1.32E+01 | –1.09E+00 |
| AP (molH^+^_eq_) | 3.62E+01 | –4.44E+00 |
| EU_T_ (molN_eq_) | 4.31E+01 | –3.53E+00 |
| EU_F_ (kgP_eq_) | 2.67E+00 | –2.97E–01 |
| EU_M_ (kgN_eq_) | 7.04E+00 | –1.34E+00 |
| E_Fw_ (CTUe) | 1.93E+05 | –1.19E+03 |
